# Prevalence of vitamin D deficiency in Africa: a systematic review and meta-analysis

**DOI:** 10.1016/S2214-109X(19)30457-7

**Published:** 2019-11-27

**Authors:** Reagan M Mogire, Agnes Mutua, Wandia Kimita, Alice Kamau, Philip Bejon, John M Pettifor, Adebowale Adeyemo, Thomas N Williams, Sarah H Atkinson

**Affiliations:** a Kenya Medical Research Institute (KEMRI), Centre for Geographic Medicine Research—Coast, KEMRI–Wellcome Trust Research Programme, Kilifi, Kenya; b Open University, KEMRI–Wellcome Trust Research Programme Affiliated Research Centre, Kilifi, Kenya; c South African Medical Research Council and Wits Developmental Pathways for Health Research Unit, Department of Paediatrics, University of the Witwatersrand, Johannesburg, South Africa; d Centre for Research on Genomics and Global Health, National Human Genome Research Institute, National Institutes of Health, Bethesda, MD, USA; e Department of Medicine, Imperial College London, London, UK; f Centre for Tropical Medicine and Global Health, Nuffield Department of Medicine, University of Oxford, Oxford, UK; g Department of Paediatrics, University of Oxford, Oxford, UK

## Abstract

**Background:**

Vitamin D deficiency is associated with non-communicable and infectious diseases, but the vitamin D status of African populations is not well characterised. We aimed to estimate the prevalence of vitamin D deficiency in children and adults living in Africa.

**Methods:**

For this systematic review and meta-analysis, we searched PubMed, Web of Science, Embase, African Journals Online, and African Index Medicus for studies on vitamin D prevalence, published from database inception to Aug 6, 2019, without language restrictions. We included all studies with measured serum 25-hydroxyvitamin D (25[OH]D) concentrations from healthy participants residing in Africa. We excluded case reports and case series, studies that measured 25(OH)D only after a clinical intervention, and studies with only a meeting abstract or unpublished material available. We used a standardised data extraction form to collect information from eligible studies; if the required information was not available in the published report, we requested raw data from the authors. We did a random-effects meta-analysis to obtain the pooled prevalence of vitamin D deficiency in African populations, with use of established cutoffs and mean 25(OH)D concentrations. We stratified meta-analyses by participant age group, geographical region, and residence in rural or urban areas. The study is registered with PROSPERO, number CRD42018112030.

**Findings:**

Our search identified 1692 studies, of which 129 studies with 21 474 participants from 23 African countries were included in the systematic review and 119 studies were included in the meta-analyses. The pooled prevalence of low vitamin D status was 18·46% (95% CI 10·66–27·78) with a cutoff of serum 25(OH)D concentration less than 30 nmol/L; 34·22% (26·22–43·68) for a cutoff of less than 50 nmol/L; and 59·54% (51·32–67·50) for a cutoff of less than 75 nmol/L. The overall mean 25(OH)D concentration was 67·78 nmol/L (95% CI 64·50–71·06). There was no evidence of publication bias, although heterogeneity was high (*I*^2^ ranged from 98·26% to 99·82%). Mean serum 25(OH)D concentrations were lower in populations living in northern African countries or South Africa compared with sub-Saharan Africa, in urban areas compared with rural areas, in women compared with men, and in newborn babies compared with their mothers.

**Interpretation:**

The prevalence of vitamin D deficiency is high in African populations. Public health strategies in Africa should include efforts to prevent, detect, and treat vitamin D deficiency, especially in newborn babies, women, and urban populations.

**Funding:**

Wellcome Trust and the DELTAS Africa Initiative.

## Introduction

Vitamin D deficiency is reported worldwide^1^ and has been associated with non-communicable and infectious diseases.^2^ Africa has a high burden of infectious diseases, and the prevalence of non-communicable diseases is increasing. A 2014 report by WHO estimates that the burden of non-communicable diseases will overtake that of communicable diseases in Africa by 2030, a trend that has been attributed to lifestyle changes related to rapid urbanisation.^3^, ^4^ Individuals of African ancestry living in temperate regions have a poorer vitamin D status than do other ethnicities, which has been associated with higher prevalence of cardiovascular disease, diabetes, and some cancers observed among African–American people.^5^ The presence of vitamin D receptors in most tissues and cells and the regulation of more than 200 human genes by vitamin D suggest that vitamin D could have diverse roles in maintaining health.^6^, ^7^

Measurement of serum 25-hydroxyvitamin D (25[OH]D) is widely accepted as a proxy for vitamin D status.^8^ However, no consensus has been reached on the definition of low vitamin D status. Rickets and osteomalacia are associated with severe vitamin D deficiency, characterised by very low concentrations of 25(OH)D, whereas extraskeletal diseases have been associated with more modest vitamin D insufficiency.^9^ Rickets and osteomalacia caused by vitamin D deficiency are considered unlikely at concentrations higher than 25 nmol/L or 30 nmol/L,^10^, ^11^ and the US National Academy of Medicine (formerly called the Institute of Medicine) recommends 25(OH)D concentrations of higher than 50 nmol/L for optimum bone health.^12^ The US Endocrine Society recommends a concentration of higher than 75 nmol/L to reduce the risk of various non-communicable and infectious diseases.^9^

**Research in context**

**Evidence before this study**

Low vitamin D status has been linked to disease. Although Africa has a high burden of disease, the prevalence of vitamin D deficiency in Africa and its association with disease has not been well characterised. Previous reviews of vitamin D status globally have reported that vitamin D deficiency exists in African populations, but these reviews had few studies from Africa and none quantified the overall prevalence. Between Sept 1, 2018, and Aug 6, 2019, we searched PubMed, Embase, Web of Science, African Journals Online, and African Index Medicus, without restriction on language or date of publication, to identify epidemiological studies that measured 25-hydroxyvitamin D (25[OH]D) concentrations in African populations.

**Added value of this study**

We estimate that approximately one in five people living in Africa have inadequate 25(OH)D concentrations (with a threshold of <30 nmol/L). The prevalence of vitamin D deficiency appears to be higher in newborn babies, urban populations, and in northern African countries and South Africa. To the best of our knowledge, this is the first systematic review and meta-analysis to quantify the prevalence of vitamin D deficiency in African populations.

**Implications of all the available evidence**

Health professionals, policy makers, and the general public in Africa should be aware of the high prevalence of vitamin D deficiency and the associated health risks. Efforts to reduce the burden of diseases in Africa should also incorporate strategies to prevent, detect, and treat vitamin D deficiency.

The prevalence of vitamin D deficiency has been estimated in temperate regions, but few prevalence studies have been done in Africa.^13^, ^14^, ^15^ We did a systematic review and meta-analysis of the prevalence of vitamin D deficiency in populations living in Africa to guide prevention, detection, and control strategies.

## Methods

### Search strategy and selection criteria

We did a systematic review and meta-analysis in accordance with the PRISMA guidelines.^16^ We searched PubMed, Embase, Web of Science, African Journals Online, and African Index Medicus for relevant articles without date or language restrictions. All the search terms were Medical Subject Heading terms, including vitamin D terms (“vitamin D”, “vitamin D deficiency”, “25-hydroxyvitamin D”, “calcifediol”, “ergocalciferols”, and “cholecalciferol”) and terms for African people and African countries (“African Continental Ancestry Group” and names of all 54 African countries). The search strategy used in PubMed was modified to suit other databases. The full search strategy is provided in the appendix 2 (p 1). We included all studies that met the inclusion criteria and that had data available before Aug 6, 2019. We also manually screened citations of relevant articles to identify additional studies.

The inclusion criteria were as follows: an original article published or accepted in a peer-reviewed journal; participants residing in Africa; a cross-sectional or longitudinal design with baseline data; and the study measured 25(OH)D in blood. We excluded studies that were conducted outside Africa; were case reports and case series; measured 25(OH)D only after a clinical intervention; or only had meeting abstract or unpublished material available. For case-control studies, only data from healthy population subgroups were considered in the meta-analyses.

We began the study selection by screening titles and abstracts of articles retrieved from the search. For articles identified to be potentially relevant, the full text was then reviewed. The full text was also reviewed if a decision could not be made from reading the title and abstract alone. Two investigators (RMM and AM)independently screened the titles and abstracts of retrieved articles and disagreements in the study selection were resolved by consensus. We quantified the inter-rater agreement for study selection using Cohen's κ coefficient.^17^ If multiple studies used the same dataset or cohort, we included the most comprehensive study with the largest number of participants and excluded the others. Studies that reported only median 25(OH)D values were excluded from meta-analyses. The study protocol is available online.

### Data analysis

Data extraction was done by two independent reviewers (RMM and WK) and compared, with disagreements resolved by discussion. We used a predefined and standardised data extraction form to collect information from all the eligible studies. All non-English-language studies were translated into English before data extraction with use of Google Translate. From each eligible study, we extracted the year of publication; first author's name; sample size; method of recruitment; study design; dates or season of blood sample collection; ethnicity; proportion of male participants; study country; method of 25(OH)D measurement; mean 25(OH)D concentrations; prevalence of vitamin D deficiency; and risk factors for low vitamin D status. If the required information was not readily available from published reports, we requested the raw data from the authors. If a study only reported 25(OH)D means for population subgroups or means for different time-points, we computed the overall mean for the cohort when appropriate. In case-control studies, only the baseline 25(OH)D levels of healthy controls were used in the meta-analysis.

We extracted data on mean 25(OH)D concentrations and the prevalence of vitamin D deficiency with use of three common cutoffs (<30 nmol/L, <50 nmol/L, and <75 nmol/L). We also collected data on factors that might influence vitamin D status, such as age, method of vitamin D measurement, area of residence (urban or rural), and geographical region.

The quality of the studies included in the meta-analysis was evaluated by a tool developed by Hoy and colleagues.^18^ Each study was assessed according to ten items and a score of one (yes) or zero (no) was assigned for each item. The studies were classified as having a low (>8), moderate (6–8), or high (≤5) risk of bias on the basis of the overall score.

All data analyses were done using R (version 3.5.1). We did meta-analyses of established cutoffs for vitamin D status (<75 nmol/L, <50 nmol/L, and <30 nmol/L) with the metaprop package,^9^, ^10^, ^12^ and a meta-analysis of mean 25(OH)D levels with the metamean package. We stratified meta-analyses by participant age group (newborn babies [<2 days old], children [2 days to 17 years], pregnant women or new mothers [mothers of newborn babies], and other adults), area of residence (urban or rural) and geographical region (northern African countries, South Africa, and sub-Saharan Africa). A random effects model was used because of high levels of heterogeneity between populations.^19^ Heterogeneity between studies was assessed using the Cochran's Q, *I*^2^, and H statistics, with an *I*^2^ of more than 75% indicating substantial heterogeneity.^20^ We explored sources of heterogeneity with a meta-regression using the metafor package. The covariates in the meta-regression included age group, geographical region, vitamin D assay, risk of bias, and area of residence. We did an influence analysis to identify outliers on the basis of a method proposed by Viechtbauer and Cheung.^21^ We did sensitivity analyses in which each of the following types of studies were excluded: studies that had fewer than 50 or 100 participants; studies with newborn babies, or pregnant women or new mothers; studies from northern African countries and South Africa; studies with a moderate or high risk of bias; studies that used assays other than the gold standard (liquid chromatography-tandem mass spectrometry); and studies published in 2009 or earlier, in 1999 or earlier, or in 1989 or earlier. The overall mean 25(OH)D concentration for each country was computed from all the eligible studies in the country, and the results were illustrated on a map of Africa using ArcGIS 10.6 (Esri, Redlands, CA, USA). To assess for publication bias, we used the Egger test of bias^22^ with p<0·05 indicating significant publication bias.

This study is registered with PROSPERO, number CRD42018112030.

### Role of the funding source

The funder of the study had no role in study design, data collection, data analysis, data interpretation, or writing of the report. The corresponding author had full access to all the data in the study and had final responsibility for the decision to submit for publication.

## Results

**Figure 1:**
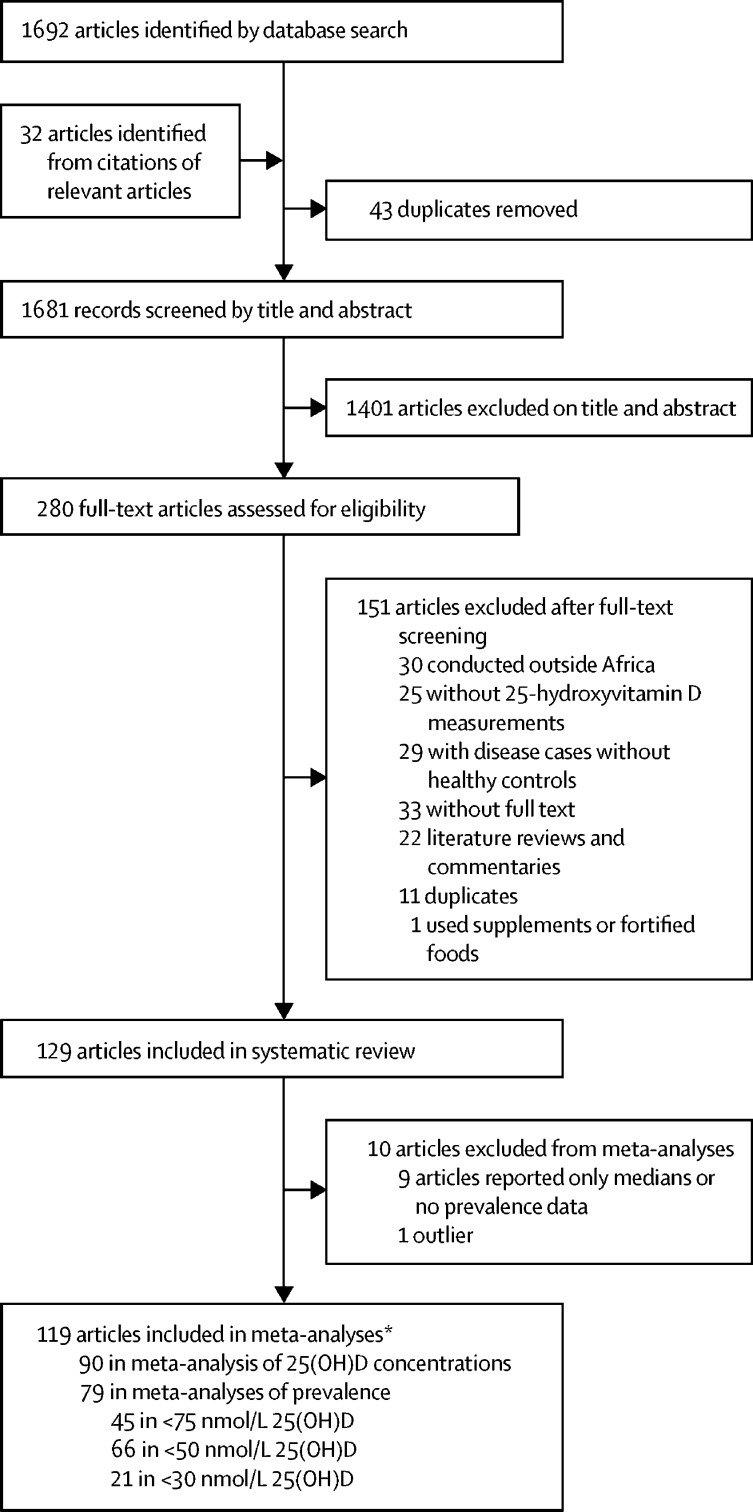
Study selection 25(OH)D=25-hydroxyvitamin D. *Some studies reported both mean 25(OH)D concentrations and prevalence of vitamin D deficiency, and some studies used more than one cutoff value.

Our search yielded 1692 articles and conference abstracts, and a further 32 articles were identified by manual screening of citations (figure 1). 43 duplicate studies were removed. After screening abstracts and titles, we excluded 1401 studies that were not relevant to our meta-analysis. After screening of full texts, we excluded an additional 151 studies that did not meet the eligibility criteria. Therefore, 129 studies with 21 474 participants from 23 African countries were included in the systematic review. 119 of these studies were included in the meta-analysis.

**Figure 2:**
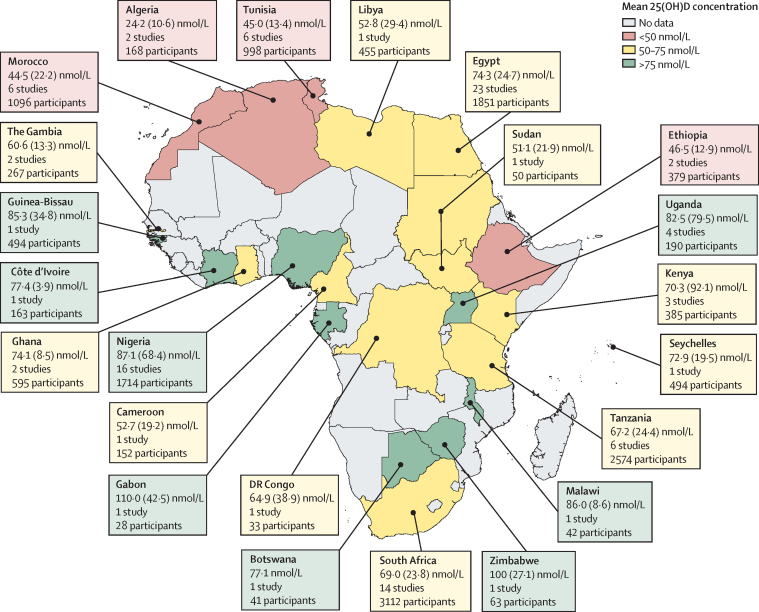
Mean 25(OH)D concentrations in African countries Data are the mean (SD) 25(OH)D concentrations reported in studies done in each country. Pooled means were calculated if the country had more than one study, and were computed only from studies that stated mean (SD) 25(OH)D concentrations. Studies that reported only median concentrations are not included in this map, with the exception of Botswana, which had a single study that reported only median levels. 25(OH)D=25-hydroxyvitamin D.

Study characteristics and their corresponding mean 25(OH)D levels are provided in the appendix 2 (pp 4–8). The studies were published between 1978 and 2019. Egypt had the highest number of eligible studies (31 studies), followed by Nigeria (21 studies) and South Africa (19 studies; figure 2, appendix 2 pp 4–8). The age of the study participants ranged from birth to 90 years and age was associated with 25(OH)D concentration in 15 (48%) of 31 studies that assessed for an association (appendix 2 p 12). 77 studies included only adult participants, 41 included only children, and 11 included both. We found no evidence of publication bias (appendix 2 p 14).

**Figure 3:**
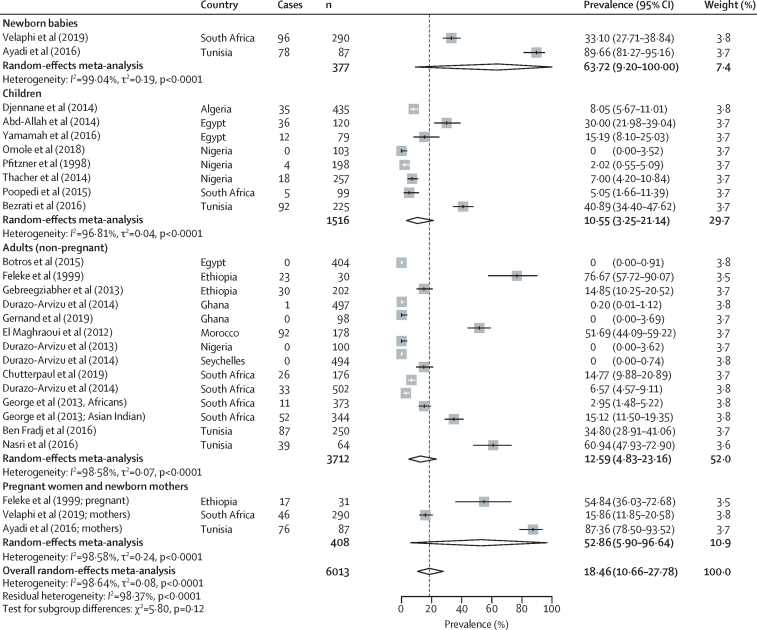
Pooled prevalence of vitamin D deficiency in Africa with use of a less than 30 nmol/L 25(OH)D cutoff Cases are defined as participants in a study with a 25(OH)D concentration of less than 30 nmol/L, and n is the total number of participants in the study. 25(OH)D=25-hydroxyvitamin D.

**Figure 4:**
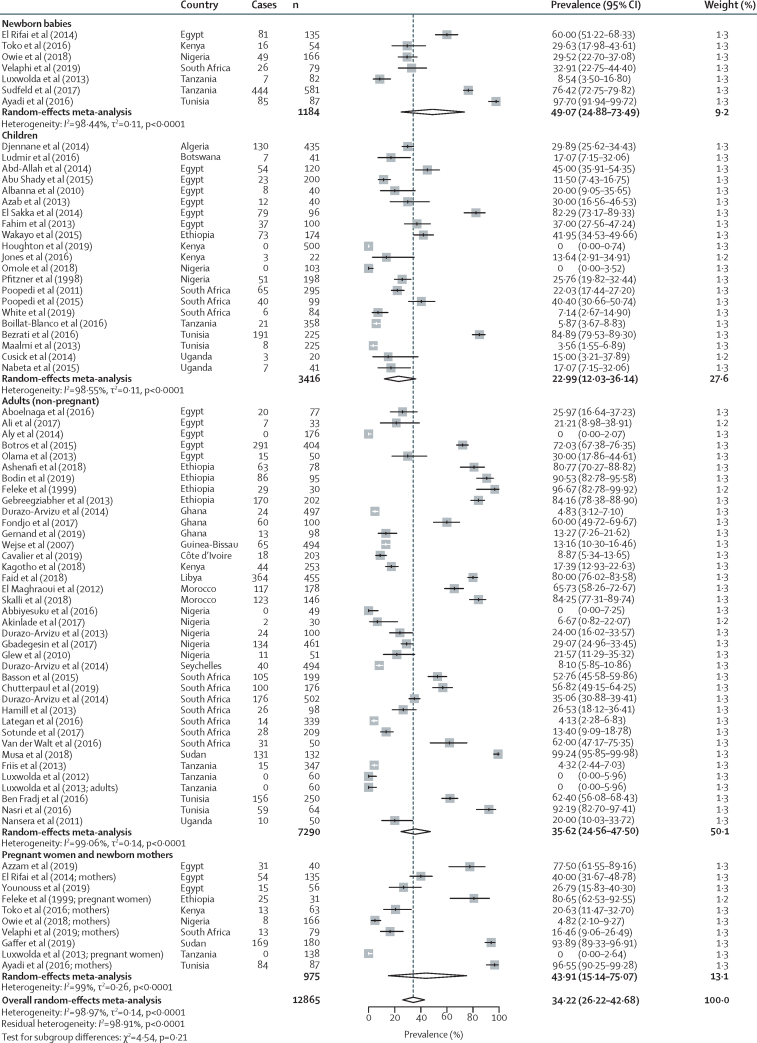
Pooled prevalence of vitamin D deficiency in Africa with use of a less than 50 nmol/L 25(OH)D cutoff Cases are defined as participants in a study with a 25(OH)D concentration of less than 50 nmol/L, and n is the total number of participants in the study. 25(OH)D=25-hydroxyvitamin D.

79 studies reported data on prespecified cutoffs for vitamin D status and were included in the meta-analyses of prevalence of low vitamin D status, with 21 studies reporting a cutoff of less than 30 nmol/L, 66 studies reporting a cutoff of less than 50 nmol/L, and 45 studies reporting a cutoff of less than 75 nmol/L. The overall prevalence of low vitamin D status was 18·46% (95% CI 10·66–27·78) for the less than 30 nmol/L cutoff (figure 3); 34·22% (26·22–43·68) for the less than 50 nmol/L cutoff (figure 4); and 59·54% (51·32–67·50) for the less than 75 nmol/L cutoff (appendix 2 pp 13). 90 studies included data on mean 25(OH)D concentration and were included in the meta-analysis of mean 25(OH)D concentration. The overall pooled mean 25(OH)D concentration was 67·78 nmol/L (95% CI 64·50–71·06); the pooled mean was 69·38 nmol/L (64·82–73·95) for adults, 65·73 nmol/L (45·65–85·81) for pregnant women and new mothers, 50·60 nmol/L (38·91–62·29) for newborn babies, and 72·22 nmol/L (64·89–79·54) for children (appendix 2 p 15).

Most studies that reported low 25(OH)D concentrations were from northern African countries and South Africa (figure 2, appendix 2 p 16). Populations in urban areas had lower vitamin D status than those in rural areas (appendix 2 pp 17, 18). Men had higher 25(OH)D concentrations than women in six (67%) of nine studies in which a comparison by sex was provided, and mothers had higher 25(OH)D concentrations than their newborn babies in all studies that included both groups (appendix 2 p 18). Case-control studies reported that children with rickets had significantly lower 25(OH)D concentrations compared with healthy community controls, and lower vitamin D status was also observed in patients with other clinical conditions compared with healthy controls (appendix 2 pp 9, 10, 18). 18 studies investigated seasonality in 25(OH)D concentrations, of which 13 (72%) reported an association (appendix 2 p 11); 12 of the 13 studies that reported seasonal differences were from northern African countries and South Africa, and one study was from sub-Saharan Africa (Tanzania).

The inter-rater agreement for study selection was high (κ=0·85, 92% agreement). Two (2%) of 129 studies were classified as having a high risk of bias, 84 (65%) were classified as having a moderate risk, and 43 (33%) were classified as having a low risk. Heterogeneity (*I*^2^) ranged from 98·26% to 99·82%, indicating substantial heterogeneity between populations, most of which could not be accounted for by age group, geographical region, residence in rural or urban settings, vitamin D assay, or risk of bias in meta-regression analyses (appendix 2 p 2).

Sensitivity analyses showed that excluding studies on the basis of age group, geographical region, risk of bias, vitamin D assay, sample size, or date of publication resulted in marginal differences in overall mean 25(OH)D concentrations (appendix 2 p 3). We identified one outlier study (by Abdel-Mohsen and colleagues^23^), which we excluded from the analyses.

## Discussion

In this systematic review and meta-analysis, we found that vitamin D deficiency, as defined by three different thresholds, is common among African populations. We found that one in five people living in Africa had a low 25(OH)D concentration with use of a less than 30 nmol/L cutoff; three in ten with use of the 50 nmol/L cutoff; and three in every five with use of the 75 nmol/L cutoff. Prevalence of vitamin D deficiency varied by region, with the highest prevalences reported in northern African countries and South Africa. Population subgroups with the lowest 25(OH)D concentrations were women, newborn babies, and urban populations. We observed substantial heterogeneity in meta-analyses estimates, which was not fully explained by age group, geographical region, residence in a rural or urban area, vitamin D assay, or risk of bias. We speculate that substantial within-population variation could exist, induced by other factors such as socioeconomic conditions, diet, custom, and coverage of skin with clothing, as previously described.^15^

The prevalence of low 25(OH)D concentrations in Africa was higher than might have been expected considering the large amounts of sunshine on the continent, and challenges the misconception that vitamin D deficiency, as defined by 25(OH)D levels of less than 30 nmol/L, is rare in Africa. Rapid urbanisation and associated lifestyle changes in Africa could explain why 25(OH)D concentrations were lower than expected.^4^ We observed that populations living in urban areas had lower 25(OH)D concentrations than rural populations, perhaps due to lifestyles that limit the duration of sunlight exposure or reduce the dietary intake of vitamin D.^24^ The UN Report on World Population Prospects estimates that more than 50% of people in Africa will live in urban areas by 2035,^4^ suggesting that the prevalence of vitamin D deficiency is likely to increase. We found that some of the studies with the highest 25(OH)D concentrations in Africa were in populations that were still practising traditional lifestyles, including nomadic animal rearing, hunting, and gathering.^25^

Of note, we found that the prevalence of vitamin D deficiency (using the <50 nmol/L cutoff) in Africa was similar to that in Europe. Nationally representative surveys in Europe found that approximately 40% of these populations have 25(OH)D concentrations of less than 50 nmol/L,^26^ compared with the prevalence of 34% that we found in Africa. Additionally, Durazo-Arvizu and colleagues^27^ observed that African people residing in Africa had similar 25(OH)D concentrations to white people residing in the USA.^27^ Prevalence of vitamin D deficiency varies globally, with reported prevalences of 23–30% in the USA,^28^, ^29^ 30–90% in the Middle East, 20% in Australia, and 56% in China.^30^, ^31^, ^32^ The large variation in vitamin D status could be accounted for by differences in known determinants of vitamin D status. For instance, supplementation and fortification of foods with vitamin D is a common source of vitamin D in North American countries and some parts of Europe,^33^, ^34^ but it is rare in Africa. Vitamin D is likely to be mostly obtained from exposure to the sun in Africa, because many of the determinants of vitamin D status in the prevalence studies included in this review were associated with sun exposure.

People of African ancestry living in temperate regions have been reported to have lower vitamin D status compared with other ethnicities in the same setting,^5^ and compared with Africans living in sub-Saharan Africa.^27^ This trend has been attributed to their skin colour being less well adapted for vitamin D synthesis in temperate climates that have less sunshine. For instance, the prevalence of vitamin D deficiency (<50 nmol/L) in African-American people living in the USA was reported to be 82·1% compared with the US national average of 41·9%.^29^ Studies have also reported a decrease in 25(OH)D concentrations in Africans with increasing distance from the equator^35^ and length of time since migrating from Africa.^36^ Similarly, we found that 25(OH)D concentrations varied by region, with the lowest concentrations observed in northern African countries and in South Africa. The vitamin D status of northern African countries was similar to populations in the Middle East, which could be attributed to similar climates and lifestyles as has been described in previous reviews.^14^, ^30^ For example, 12 of 13 studies that reported an effect of seasonality on vitamin D status were from northern African countries and South Africa; a seasonality trend is common in temperate regions because of distinct seasons of the year with variable sunshine hours.

Several other factors could be affecting vitamin D status in Africa. In subgroup analyses, we found that vitamin D status varied by age in African populations, with the lowest 25(OH)D concentrations observed in newborn babies. A systematic review reported that 25(OH)D concentrations were lower in newborn babies than their mothers and that concentrations were highly correlated between newborn babies and their mothers.^37^ In the three studies that included populations from both urban and rural areas in Africa, participants from urban areas had lower 25(OH)D concentrations than those in rural areas (appendix 2 p 18).^27^ In agreement with studies from other populations,^14^ we found that women living in Africa tended to have lower 25(OH)D concentrations than men in most studies. Meta-analysis of prevalence results showed that pregnant women and new mothers had a higher prevalence of vitamin D deficiency (<50 nmol/L) than other adults (44% *vs* 36%), a trend that was mostly observed in northern African countries. During pregnancy, 25(OH)D concentrations are expected to increase to ensure that the fetus receives sufficient calcium for growth and development.^37^, ^38^

The prevalence of rickets is high in Africa, although could be caused by calcium deficiency rather than vitamin D deficiency in some populations.^15^ Some African populations have been reported to have some of the lowest dietary intakes of calcium globally, which is concerning because calcium deficiency is an important cause of rickets in Africa, particularly in combination with poor vitamin D status.^15^, ^39^ All of the case-control studies included in this review reported that children with rickets had lower 25(OH)D concentrations compared with healthy community controls (appendix 2 p 18), suggesting that vitamin D deficiency could also be important in the pathology of rickets in Africa. Most of the clinical illnesses investigated in this review were associated with lower vitamin D status in cases compared with control groups (appendix 2 pp 9, 10) and many pathways and mechanisms of action have been suggested by which vitamin D could affect susceptibility to disease.^7^ However, the studies included in this review were observational and could not provide evidence of causality.

To the best of our knowledge, this is the first meta-analysis of the prevalence of vitamin D deficiency and mean 25(OH)D concentrations in the general population in Africa and includes the largest number of studies from Africa. However, our findings should be interpreted in the context of some limitations. Three studies included in the meta-analyses were published before 1990 and might not be representative of current vitamin D status, although sensitivity analyses revealed that excluding these studies resulted in only marginal changes in the overall estimate of mean 25(OH)D concentration. In addition, many African countries did not have any studies that measured vitamin D status, and more studies are needed to better reflect heterogeneity in African populations. A more detailed analysis of the factors associated with vitamin D status could have been done with access to individual-level datasets, rather than relying on published summary measures. Studies included in this review used different vitamin D assays, which might have influenced our findings; recalibration of studies, as was previously done in Europe,^26^ might provide a better representation of vitamin D status. However, sensitivity analyses showed that assay type did not have a significant effect on the overall estimate of mean 25(OH)D concentration and contributed to only about 5% of observed heterogeneity. Although we only included studies with healthy participants in this review, population-based studies are better at inferring the vitamin D status of the general population, and few studies of this type have been done in Africa.

In conclusion, we found that vitamin D deficiency, as defined by three different thresholds, is prevalent in Africa, particularly in newborn babies, women, urban populations, and populations living in northern African countries and South Africa. Strategies to prevent, detect, and treat vitamin D deficiency need to be incorporated into public health and primary care in Africa. Therefore, we recommend the development of governmental policies and nutritional guidelines to improve vitamin D status, and dietary calcium intakes when appropriate, of African populations as has been done in other continents and countries.

## Supplementary Materials

Kiswahili translation of the abstract
