# Correction to *Lancet Glob Health* 2020; 8: e134–42

**DOI:** 10.1016/S2214-109X(22)00083-3

**Published:** 2022-03-15

*Mogire RM, Mutua A, Kimita W, et al. Prevalence of vitamin D deficiency in Africa: a systematic review and meta-analysis.* Lancet Glob Health *2020; **8:** e134–42*—In this Article, some data were misclassified by subgroup in the meta-analysis and an article from the Seychelles (Laird et al, 2017) was omitted from the meta-analysis. The estimates for the prevalence of vitamin D deficiency and mean vitamin D concentration have been adjusted as a result of these corrections, but the changes had no effect on the conclusions of the study. Further detail of the corrections is provided in the accompanying Correspondence.^1^, ^2^ The corrections have been made as of March 15, 2022.
