# Supplementary material for: Prevalence of vitamin D deficiency in Africa: a systematic review and meta-analysis
Source: Lancet Glob Health. 2019 Nov 27;8(1):e134–42. doi: 10.1016/S2214-109X(19)30457-7 (PMC7024961; doi:10.1016/S2214-109X(19)30457-7)
Supplement: Kiswahili translation of the abstract [file mmc1.pdf]

THE LANCET
Global Health

Supplementary appendix

This translation in Kiswahili was submitted by the authors and we reproduce it as supplied. It has not been peer reviewed. *The Lancet Global Health*'s editorial processes have only been applied to the original in English, which should serve as reference for this manuscript.

Supplement to: Mogire RM, Mutua A, Kimita W, et al. Prevalence of vitamin D deficiency in Africa: a systematic review and meta-analysis. *Lancet Glob Health* 2019; published online Nov 27. http://dx.doi.org/10.1016/S2214-109X(19)30457-7.

Tafsiri hii katika Kiswahili iliwasilishwa na waandishi na tunatengeneza tena kama hutolewa. Haijapitiwa. Mchakato wa hariri wa *Lancet Global Health* umetumika tu kwa asili kwa Kiingereza, ambayo inapaswa kutumika kama kumbukumbu kwa muswada hii.

Imeongeza kwa: Mogire RM, Mutua A, Kimita W, et al. Upungufu wa Vitamini D barani Afrika. *Lancet Glob Health* 2019; iliyochapishwa mtandaoni Novemba 27. http://dx.doi.org/10.1016/S2214-109X(19)30457-7.

Upungufu wa Vitamini D barani Afrika

Ikisiri

Utangulizi: Upungufu wa vitamini D umeathiri watu wengi duniani, hili ni jambo linaloleta wasiwasi kwa sababu upungufu huu umehusishwa na magonjwa mbali mbali. Mengi ya magonjwa haya yanapatikana barani Afrika. Walakini, upungufu wa vitamini D katika bara la Afrika haujachunguzwa kwa undani. Lengo letu lilikuwa kukadiria idadi ya watu waliokuwa na upungufu wa vitamini D katika bara la Afrika.

Mbinu: Tulitafuta makala yote ya kisayansi yaliyohifadhiwa kwenye hifadhidata za PubMed, Web of Science, Embase, African Journals Online na African Index Medicus yaliyoangazia upungufu wa vitamini D katika bara la Afrika. Tulijumuisha miradi yote yaliyopima viwango vya vitamini D ya wakaazi wa Afrika wasio wagonjwa bila kuzingatia mwaka au lugha ya uchapishaji wa makala hayo. Tulikadiria idadi ya watu walio na upungufu wa vitamini D kutumia viwango vitatu tofauti vinavyotumika duniani: <75 nmol/L, <50 nmol/L, na <30 nmol/L. Tulifanya uchambuzi huu tukizingatia umri (watoto kwa watu wazima), jinsia (wake kwa waume), maeneo ya kijiografia ya Afrika (Afrika mashariki, Afrika ya Kati, Afrika Magharibi, Afrika Kaskazini, na Afrika Kusini), na makazi (vijijini au mijini).

Matokeo: Utafiti huu ulijumuisha miradi 133 ya kisayansi yaliyoshirikisha watu 21,591 kutoka nchi 23 za Kiafrika. Kwa ujumla, matokeo ya uchambuzi huu yanadhihirisha kuwa takriban asilimia 58·8 ya wakaazi wa Afrika walikuwa na vitamini D chini ya 75 nmol/L, asilimia 34·4 chini ya 50 nmol/L, na asilimia 18·5 chini ya 30 nmol/L. Makundi yaliyoathirika zaidi yalikuwa ya watoto wachanga, wanawake, wanaoishi mbali na ikweta na wakaazi wa mijini. Watu wengi wenye upungufu wa vitamini D walikuwa kutoka nchi za Afrika Kusini na Kaskazini. Tulikadiria kiwango cha wastani ya vitamini D ya wakaazi wa Afrika kuwa 64·6 nmol/L.

Hitimisho: Matokeo ya utafiti huu yanaashiria kuwa upungufu wa vitamini D umeenea barani Afrika. Tunapendekeza kujumuisha juhudi za kuzuia, kugundua na kutibu upungufu wa vitamini D katika mikakati ya kuboresha afya ya umma, haswa katika makundi yaliyoathirika zaidi.
