# Supplementary material for: Prevalence of vitamin D deficiency in Africa: a systematic review and meta-analysis
Source: Lancet Glob Health. 2019 Nov 27;8(1):e134–42. doi: 10.1016/S2214-109X(19)30457-7 (PMC7024961; doi:10.1016/S2214-109X(19)30457-7)
Supplement: Supplementary appendix 2 [file mmc2.pdf]

THE LANCET
Global Health

Supplementary appendix

This appendix formed part of the original submission and has been peer reviewed. We post it as supplied by the authors.

Supplement to: Mogire RM, Mutua A, Kimita W, et al. Prevalence of vitamin D deficiency in Africa: a systematic review and meta-analysis. *Lancet Glob Health* 2019; published online Nov 27. http://dx.doi.org/10.1016/S2214-109X(19)30457-7.

Appendix 2

Supplementary Table 1. Keywords used to search databases.

Database	Search terms
PubMed/ MEDLINE*	("Vitamin D"[Mesh] OR "Vitamin D Deficiency"[Mesh] OR "Cholecalciferol"[Mesh] OR "25-Hydroxyvitamin D 2"[Mesh] OR "Calcifediol"[Mesh] OR "Ergocalciferols"[Mesh]) AND ("African Continental Ancestry Group"[Mesh] OR “Algeria”[Mesh] OR “Egypt”[Mesh] OR “Libya”[Mesh] OR “Morocco”[Mesh] OR “South Sudan”[Mesh] OR “Sudan”[Mesh] OR “Tunisia”[Mesh] OR “Burundi”[Mesh] OR “Comoros”[Mesh] OR “Djibouti”[Mesh] OR “Eritrea”[Mesh] OR “Ethiopia”[Mesh] OR “Kenya”[Mesh] OR “Madagascar”[Mesh] OR “Malawi”[Mesh] OR “Mauritius”[Mesh] OR “Comoros”[Mesh] OR “Mozambique”[Mesh] OR “Reunion”[Mesh] OR “Rwanda”[Mesh] OR “Seychelles”[Mesh] OR “Somalia”[Mesh] OR “Tanzania”[Mesh] OR “Uganda”[Mesh] OR “Zambia”[Mesh] OR “Zimbabwe”[Mesh] OR “Benin”[Mesh] OR “Burkina Faso”[Mesh] OR “Cabo Verde”[Mesh] OR “Cote d'Ivoire”[Mesh] OR “Gambia”[Mesh] OR “Ghana”[Mesh] OR “Guinea”[Mesh] OR “Guinea-Bissau”[Mesh] OR “Liberia”[Mesh] OR OR “Mali”[Mesh] OR “Mauritania”[Mesh] OR “Niger”[Mesh] OR “Nigeria”[Mesh] OR “Atlantic Islands”[Mesh] OR “Senegal”[Mesh] OR “Sierra Leone”[Mesh] OR “Togo”[Mesh] OR “Angola”[Mesh] OR “Cameroon”[Mesh] OR “Central African Republic”[Mesh] OR “Chad”[Mesh] OR “Congo”[Mesh] OR “Democratic Republic of the Congo”[Mesh] OR “Equatorial Guinea”[Mesh] OR “Gabon”[Mesh] OR “Sao Tome and Principe”[Mesh] OR “Botswana”[Mesh] OR “Lesotho”[Mesh] OR “Namibia”[Mesh] OR “South Africa”[Mesh] OR “Swaziland”[Mesh])
Web of Science	ts = (" vitamin D" and ("Algeria" or " Egypt" or " Libya" or " Morocco" or " South Sudan" or " Sudan" or " Tunisia" or " Western Sahara" or " Burundi" or " Comoros" or " Djibouti" or " Eritrea" or " Ethiopia" or " Kenya" or " Madagascar" or " Malawi" or " Mauritius" or "Comoros" or " Mozambique" or " Reunion" or " Rwanda" or " Seychelles" or " Somalia" or " Tanzania" or " Uganda" or " Zambia" or " Zimbabwe" or " Benin" or " Burkina Faso" or " Cape Verde" or " Cote d'Ivoire " or " Ivory Coast" or " Gambia" or " Ghana" or " Guinea" or " Guinea-Bissau" or " Liberia" or " Mali" or " Mauritania" or " Niger" or " Nigeria" or "Atlantic Islands" or " Senegal" or " Sierra Leone" or " Togo" or " Angola" or " Cameroon" or " Central African Republic" or " Chad" or " Congo" or " Democratic Republic of the Congo" or " Equatorial Guinea" or " Gabon" or " Sao Tome and Principe" or " Botswana" or " Lesotho" or " Namibia" or " South Africa" or " Swaziland")
Embase	((" vitamin D" and ("Algeria" or " Egypt" or " Libya" or " Morocco" or " South Sudan" or " Sudan" or " Tunisia" or " Western Sahara" or " Burundi" or " Comoros" or " Djibouti" or " Eritrea" or " Ethiopia" or " Kenya" or " Madagascar" or " Malawi" or " Mauritius" or " Comoros" or " Mozambique" or " Reunion" or " Rwanda" or " Seychelles" or " Somalia" or " Tanzania" or " Uganda" or " Zambia" or " Zimbabwe" or " Benin" or " Burkina Faso" or " Cape Verde" or " Cote d'Ivoire " or " Ivory Coast" or " Gambia" or " Ghana" or " Guinea" or " Guinea-Bissau" or " Liberia" or " Mali" or " Mauritania" or " Niger" or " Nigeria" or "Atlantic Islands" or " Senegal" or " Sierra Leone" or " Togo" or " Angola" or " Cameroon" or " Central African Republic" or " Chad" or " Congo" or " Democratic Republic of the Congo" or " Equatorial Guinea" or " Gabon" or " Sao Tome and Principe" or " Botswana" or " Lesotho" or " Namibia" or " South Africa" or " Swaziland")) not "African American").mp. [mp=title, abstract, heading word, drug trade name, original title, device manufacturer, drug manufacturer, device trade name, keyword, floating subheading word, candidate term word]
African Journals OnLine (AJOL)	“vitamin D" and ("Algeria" or "Egypt" or "Libya" or "Morocco" or "South Sudan" or "Sudan" or "Tunisia" or "Western Sahara" or "Burundi" or "Comoros" or "Djibouti" or "Eritrea" or "Ethiopia" or "Kenya" or "Madagascar" or "Malawi" or “Mauritius" or " Mayotte" or " Mozambique" or " Reunion" or " Rwanda" or " Seychelles" or " Somalia" or " Tanzania" or " Uganda" or " Zambia" or " Zimbabwe" or " Benin" or " Burkina Faso" or " Cape Verde" or " Cote d'Ivoire " or " Ivory Coast" or " Gambia" or " Ghana" or " Guinea" or " Guinea-Bissau" or " Liberia" or " Mali" or " Mauritania" or " Niger" or " Nigeria" or "Atlantic Islands" or " Senegal" or " Sierra Leone" or " Togo" or " Angola" or " Cameroon" or vitamin D" and (" Central African Republic" or " Chad" or " Congo" or " Democratic Republic of the Congo" or " Equatorial Guinea" or " Gabon" or " Sao Tome and Principe" or " Botswana" or " Lesotho" or " Namibia" or " South Africa" or " Swaziland") not "African American”
African Index Medicus	“vitamin D”

*Country names were also searched without their “Mesh” terms in PubMed.

Supplementary Table 2. Sources of heterogeneity assessed using meta-regression analyses (mean 25(OH)D levels in 107 studies)

Factors	Number of studies (%)	Pooled 25(OH)D mean (*nmol/L*)	95% Cl	P value	Residual I^{2*}	Percentage change in I^2
**Age groups**						
• Newborns	11 (10%)	50·8	35·4, 66·3	0·12	99·82%	9%
• Children	29 (27%)	72·3	38·7, 105·9			
• Non-pregnant adults	56 (52%)	69·3	36·9, 101·6			
• Pregnant/newborn mothers	12 (11%)	65·8	28·9, 102·6			
**Geographical Region**						
• Sub-Saharan Africa (West, East and Central Africa)	52 (49%)	74·30	56·0, 92·60	0·025	99·90%	1%
• North Africa	42 (39%)	59·7	51·9, 67·5			
• South Africa	16 (15%)	68·2	44·5, 91·9			
**Setting**						
• Rural	16 (29%)	74·4	62·6, 86·1	0·26	99·90%	1%
• Urban	54 (71%)	66·7	41·5, 91·8			
**Assay**						
• CLIA	23 (26%)	60·0	50·2, 69·8	0·018	99·86%	5%
• CPBA	4 (4%)	60·8	25·4, 96·1			
• EIA	5 (6%)	86·2	52·9, 119·4			
• ELISA	22 (21%)	66·5	42·6, 90·4			
• HPLC	10 (9%)	84·6	56·9, 112·2			
• LC–MS/MS	10 (9%)	79·9	52·3, 107·5			
• RIA	23 (21%)	59·6	35·9, 83·3			
**Risk of bias**						
• High risk of bias	2 (2%)	24·2	-11·4, 59·8	0·039	99.89%	2%
• Moderate risk of bias	67 (62%)	71·3	-0·8, 143·4			
• Low risk of bias	39 (36%)	67·1	-4·7, 138·8			

*I^2, measure of heterogeneity. Summary unadjusted I^2 = 99·91%; Percentage change in I^2 computed as: (Summary unadjusted I^2 - Residual I^2) / Summary unadjusted $I^2 \times 100$

Supplementary Table 3. Sensitivitiy analyses. Meta-analysis of mean 25(OH)D levels excluding specific study groups

Study category excluded	Number of studies (%)	Pooled 25(OH)D mean (*nmol/L*)	95% CI	I^{2*}
**All studies (none excluded)**	107 (100%)	67·8	62·8, 72·8	99·91%
**Excluding age groups;**				
• Newborns	11 (10%)	69·7	64·6, 74·9	99·87%
• Pregnant mothers	12 (11%)	68·1	62·9, 73·2	99·88%
• Both pregnant mothers and newborns	23 (21%)	70·3	65·0, 75·6	99·83%
**Excluding African region;**				
• Northern African countries	42 (39%)	72·7	62·8, 72·8	99·92%
• Southern African countries	16 (14%)	67·0	61·4 72·6	99·92%
• Both Northern and Southern African countries	58 (53%)	72·8	65·6, 80·1	99·94%
**Excluding studies with high risk of bias;**				
• High risk of bias	2 (2%)	68·6	63·7, 73·6	99·91%
• Moderate risk of bias	66 (62%)	70·0	60·4, 77·6	99·95%
• Both High and moderate risk of bias	68 (64%)	71·3	62·9, 79·7	99·94%
**Excluding studies with the following assays;**				
• CLIA	23 (26%)	69·6	63·9, 75·3	99·92%
• CPBA	4 (4%)	67·7	62·5, 72·8	99·91%
• EIA	5 (6%)	66·4	61·2, 71·5	99·91%
• ELISA	22 (21%)	67·6	61·9, 73·4	99·82%
• HPLC	10 (9%)	65·4	60·3, 70·4	99·89%
• RIA	23 (21%)	69·8	63·7, 75·9	99·92%
**Excluding studies with;**				
• n < 50	35 (32%)	70·4	62·0, 78·8	99·18%
• n < 100	61 (57%)	66·7	60·0, 73·5	99·74%
**Excluding studies published between;**				
• 1978 to 1989	3 (3%)	67·9	62·8, 73·1	99·91%
• 1978 to 1999	14 (13%)	70·3	64·9, 75·8	99·91%
• 1978 to 2009	24 (22%)	70·6	64·7, 76·4	99·92%

$^{*}I^{2}$, measure of heterogeneity ³Vitamin D measurement assays; RIA: Radioimmunoassay; CLIA: Chemiluminescence immunoassays; HPLC–MS/MS high-performance liquid chromatography-tandem mass spectrometry; HPLC: high-performance liquid chromatography; EIA: Enzyme immunoassay; CPBA: competitive protein-binding assay.

Supplementary Table 4. Summary characteristics of eligible studies (in chronological order)

1st Author, year	City, country	Eligible subjects (n)	Male (%)*	Mean age (range/SD) in years*	Area of residence	Vitamin D measurement method³	Vitamin D mean (SD) / median (IQR) (nmol/L)€
**Children (≤ 17 Years)**							
Velaphi 2019[1] (newborns)	Johannesburg, South Africa	290	0%	0	Urban	CLIA	57·0 (29·7)
Hassam 2019[2]	Dar es Salaam Tanzania	47	53%	1	NA	EIA	47·79
Houghton 2019[3]	Emali, Kenya	Kamba: 287<br>Maasai: 213	50%	4 (3–5)	Rural	LC–MS/MS	Kaamba : 82·5 (80·7, 84·3)<br>Maasai: 95·7 (93·1, 98·3)
Bodin 2019[4]	Addis Ababa, Ethiopia	95	50%	4·3	Urban	HPLC–MS/MS	35·2
White 2019[5]	Pretoria, South Africa	84	48%	8·5	Urban	LC–MS/MS	68·1 (13·2)
Owie 2018[6] (newborns)	Lagos, Nigeria	166	NA	0 (0)	Urban	ELISA	63·4 (1·5)
Youssef 2018[7] (newborns)	Egypt	56	0%	NA	NA	EIA	NA
Omole 2018[8]	Ilesa, Nigeria	103	64·1%	6·6 (7–14)	NA	HPLC	127·8 (17·2)
Sudfeld 2017[9]	Daresalaam, Tanzania	581	48%	0·3 (0·1–0·5)	Urban	HPLC–MS/MS	1·5 months: 36·2 (18·5)<br>6 months: 64·9 (21·7)
Ayadi 2016[10] (newborns)	Tunis, Tunisia	87	45%	0 (0)	Rural and urban	CLIA	14·8 (10·4)
Jones 2016[11]	Nairobi, Kenya	22	64%	1·1 (0·8–1·5)	Urban	CLIA	70 (54–85)
Bezrati 2016[12]	Tunis, Tunisia	225	NA	11 (7–16)	Urban	CLIA	35·0 (12·7)
Yamamah 2016[13]	South Sinai, Egypt	79	52%	8·6 (4–12)	Rural	ELISA	NA
Toko 2016[14] (newborns)	Chulaimbo, Kenya	54	57%	0 (0)	Rural	EIA	64·9 (26·4)
Boillat-Blanco 2016[15]	Kinondoni, Tanzania	358	53%	0·5	NA	CLIA	89·6 (26·9)
Ludmir 2016[16]	Gaborone, Botwsana	41	41%	1	NA	CLIA	77·1
Eltayeb 2015[17]	Assyout, Egypt	28	65%	9·35 (7–14)	Urban	ELISA	98·31 (3·5)
Braithwaite 2015[18]	West Kiang, Gambia	237	37%	11·9 (17–19)	Rural	CLIA	59·6 (12·9)
Sudfeld 2015[19]	Daresalaam, Tanzania	948	54%	0·1 (0·1–0·1)	Urban	HPLC–MS/MS	45·2 (23)
Poopedi 2015[20]	Johannesburg, South Africa	99	58%	15 (11–20)	Urban	CLIA	56·4 (7·2)
Wakayo 2015[21,22]	Ethiopia	174	43%	15 (11–18)	Rural and urban	CLIA	54·5 (15·9)
Abu Shady 2015[23]	Egypt	200	49%	10 (9–11)	NR	ELISA	103·7 (33·2)
Nabeta 2015[24]	Kampala, Uganda	41	54%	1·3 (0·5–2·0)	Urban	CLIA	80·4 (27·2)
Cusick 2014[25]	Uganda	20	45%	3 (1–12)	Urban	CLIA	63·1 (21·7)
Djennane 2014[26]	Algeria	435	47%	10 (5–15)	Urban	CLIA	Summer: 71·4 (48·2–79·5)<br>Winter: 52·9 (39·4–75·6)
El Rifai 2014[27] (newborns)	Cairo, Egypt	135	53%	0 (0)	Rural and urban	ELISA	41·7 (25·0)
El Sakka 2014[28]	Egypt	96	58%	1 (0·25)	NA	RIA	NA
Abd–Allah 2014[29]	Zagazig, Egypt	120	40%	11 (7–17)	Urban	ELISA	46·6 (13·5)
Fares 2014[30]	Tunis, Tunisia	156	51%	0	Urban	RIA	29·8 (15·2)

Thacher 2014[31]	Jos, Nigeria	257	50%	(1–10)	Urban	LC–MS/MS	49·9 (7·5–127·3)
Maalmi 2013[32]	Ariana, Tunisia	225	56%	9·5 (2–16)	Rural	RIA	75·6 (14·9)
Azab 2013[33]	Zagazig, Egypt	40	43%	10·8 (6–16)	Urban	ELISA	66·1 (12)
Fahim 2013[34]	Assiut, Egypt	100	NA	8 (4–15)	NA	ELISA	40·2 (12·3) pg/ml
Luxwolda 2013[35] (newborns)	Tanzania	82	60%	0	Rural	LC–MS/MS	79·0 (26·4)
Amukele 2012[36]	Malawi	21	NA	0 (0–1)	NA	LC–MS/MS	78·6 (10·4)
Hamza 2011[37]	Cairo, Egypt	60	17%	13·10 (7·2–18·5)	Urban	ELISA	106·5 (23)
Poopedi 2011[38]	Johannesburg, South Africa	295	78%	10·5	Urban	CLIA	93·4 (32·8)
Thacher 2010[39]	Jos, Nigeria	21	48%	3 (2–5)	Urban	CLIA	67
Albanna 2010[40]	Zagazig, Egypt	40	50%	3 (2–5)	Urban	EIA	87·25 (18·4)
Nguema - Asseko 2005[41]	Oyem, Gabon	28	56%	0 (0)	Urban	NA	110·0 (42·5)
Graff 2004[42]	Jos, Nigeria	15	40%	4 (2–8)	Urban	CPBA	72·4 (11·5)
Thacher 2000[43,44]	Jos, Nigeria	123	50%	4 (2–6)	Urban	RIA	51·2 (15·5)
Thacher 1999a[45]	Jos, Nigeria	10	NA	7 (1–8)	Urban	RIA	52·2 (7·2)
Pfitzner 1998[46]	Jos, Nigeria	198	45%	2·0 (0·5–3·0)	Urban	RIA	64·9 (24)
Cornish 2000[47]	N· Province, South Africa	58	NA	12	Rural	RIA	111·4 (9·1)
Walter 1997[48]	Jos, Nigeria	27	70%	3 (1–7)	Urban	RIA	59·9 (18·7)
Sanchez 1997[49] (newborns)	Maiduguri, Nigeria	10	0%	0	NA	RIA	32·9 (19·3)
Oginni 1996a[50]	Ile-Ife, Nigeria	94	63%	3 (1–5)	Urban	RIA	63 (2·6)
Oginni 1996b[51]	Ile-Ife, Nigeria	20	61%	3 (1–5)	Urban	RIA	69 (22)
Okonofua 1991[52]	Ile-Ife, Nigeria	12	75%	2	Urban	RIA	41 (29–50)
Okonofua 1986[53] (newborns)	Ife, Nigeria	30	NA	0	NA	CPBA	49 (12·8)
Markestad 1984[54] (newborns)	Libya	14	NA	0	NA	NA	20 (10–45)
**Adults (≥18 years)**							
Velaphi 2019[1] (mothers)	Johannesburg, South Africa	290	NA	NA	Urban	CLIA	41·9 (21·0)
Oluwole 2019[55]	Lagos, Nigeria	206	0%	31	Urban	ELISA	142·3 (112·3–164·7)
Azzam 2019[56]	Alexandria, Egypt	40	0%	NA	NA	ELISA	40·3 (21·4)
Cavalier 2019[57]	Abidjan, Ivory Coast	203	40%	37	Urban	CLIA	64·6
Chikwati 2019[58]	Harare, Zimbabwe	63	0%	26·6 (18–49)	Urban	HPLC	100 ± 27·1
Chutterpaul 2019[59]	KwaZulu-Natal, South Africa	176	16·5%	72·6	NA	HPLC	50·1 ± 23·3
Gaffer 2019[60]	Khartoum, Sudan	180	0%	27·7	Rural	CLIA	28·2 (23·5,36·2)
Gernand 2019[61]	Asesewa, Ghana	98	0%	26·5 (18–35)	Rural	LC–MS/MS	65·1 (14·2)
Younouss 2019[62]	Yaounde (Cameroon	75	0%	27·6	NA	ELISA	46·1
Owie 2018[6] (mothers)	Lagos, Nigeria	166	NA	31·4 (18–42)	Urban	ELISA	87·4 (2·0)

Myburgh 2018[63]	NW Province, South Africa	505	0%	NA	Rural and urban	CLIA	68·2 (median)
Kagotho 2018[64]	Nairobi, Kenya	253	75%	33 (18–65)	Urban	CLIA	69·4 (111·8)
Faid 2018[65]	Misurata, Libya	455	16%	33 (1–64)	Urban	CLIA	52·8 (29·4)
Ibrahim 2018[66]	Qena, Egypt	20	50%	35·2	NA	ELISA	76·7 (10·73)
Ashenafi 2018[67]	Addis Ababa, Ethiopia	78	54%	29 (18–68)	Urban	NA	35
Skalli 2018[68]	Rabat, Morocco	146	NA	33·6 (18–60)	Urban	LC–MS/MS	32·4 (16·4)
Lahmar 2018[69]	Ariana, Tunisia	154	43%	46·36	NA	RIA	70·3 (11·7)
Aké 2018[70]	Côte d'Ivoire	163	NA	18–49	NA	HPLC	77·4 (3·9)
Bahendeka 2018[71]	Uganda·	79	33%	17·3 (7·3–27·9)	NA	CLIA	99·6· (120·8)
Bakeer 2018[72]	Egypt	17	0%	26·2 (19–35)	NA	ELISA	48·65 (27·30)
Youssef 2018[7] (mothers)	Egypt	56	0%	NA	NA	EIA	NA
Fondjo 2017[73]	Nkawie, Ghana	100	22%	57·7	Rural	ELISA	31·3 (6·5–81·8)
Ali 2017[74]	Cairo, Egypt	33	42%	35 (27–59)	Urban	ELISA	90·1 (26·9–189·1)
Musa 2018[75]	Khartoum, Sudan	132	0%	27·6	Rural and urban	CLIA	21·0 (18·0–27·7)
Abdel Galil 2018[76]	Zagazig, Egypt	100	0%	36·24	Urban	ELISA	114·4 (22·9)
Bakeer 2018[72]	Egypt	17	0%	26 (19–35)	NA	ELISA	48·7 (27·3)
Abdel–Mohsen 2018[53]	Alexindiria, Egypt	30	NA	NA	Urban	ELISA	164·7 (7·5)
Sotunde 2017[77]	NW Province, South Africa	209	0%	60 (43–80)	Urban	CLIA	76·4 (23·4)
Akinlade 2017[78]	Idadan, Nigeria	30	NA	35 (19–55)	NA	ELISA	28·1 (5·6)
Gbadegesin 2017[79]	Lagos, Nigeria	461	0%	31·3 (4·4)	NA	HPLC	130·3 (129·7)
Derar 2017[80]	Khartoum, Sudan	50	NA	32 (18–60)	NA	NA	51·1 (21·9)
Fondjo 2017[73]	Nkawie, Ghana	100	22%	58	Rural	ELISA	31·3 (6·5,81·8)
Musarurwa 2017[81]	Harare, Zimbabwe	133	42%	35·1 (8·9)	NA	CLIA	53·9 (45·7,66·1)
Nielson 2016[82]	Keneba, The Gambia	17	–	29 (25–39)	Rural	EIA	20·1 (5·8) pM
Lategan 2016[83]	Mangaung, South Africa	339	22%	44 (25–63)	Urban	CLIA	96·8 (28)
Ayadi 2016[10] (mothers)	Tunis, Tunisia	87	0%	31 (19–42)	Rural and urban	CLIA	17·0 (12·8)
El Maataoui 2016[84]	Morocco	254	27%	60·5 (8·4)	NA	CLIA	50·6 (21·5)
Edem 2016[85]	Ibadan, Nigeria	20	NA	NA	Urban	HPLC	45·80 (13·30) pg/ml
Abbiyesuku 2016[86]	Ibadan, Nigeria	49	100%	54·50 (30–80)	Urban	HPLC	107·2 (25·2)
Toko 2016[14] (mothers)	Chulaimbo, Kenya	63	0%	22·5	Rural	EIA	77·0 (31·5)
Aboelnaga 2016[87]	Dakalia, Egypt	50	36%	39·5 (18–65)	Rural	ELISA	60·4 (21·7)
Van der Walt 2016[88]	Free State province, South Africa	50	33%	39	Rural and urban	ELISA	45·9
Azab 2016[89]	Zagazig, Egypt	100	5%	11·5 (8–18)	Urban	ELISA	84·1 (3·7)

Ben Fradj 2016[90]	Tunis, Tunisia	250	25%	63·3 (29–91)	Urban	CLIA	NA
Nasri 2016[91]	Tunis, Tunisia	64	0%	NA	Urban	CLIA	28·3 (13·82)
Sobeih 2016[92]	Cairo, Egypt	75	NA	31·5 (14–65)	Urban	ELISA	71·9 (26·2)
Basson 2015[93]	Cape Town, SA	199	31%	34·0 (24·0–44·3)	Rural and urban	CLIA	49·2 (42·4–59·7)
Fattah 2015[94]	Cairo, Egypt	30	60%	25·1 (19–50)	Urban	EIA	112·8 (51·2)
Botros 2015[95]	Cairo, Egypt	404	0%	31·5 (8·2)	NA	RIA	27·5 (4·0–62)
El Maataoui 2015[96]	Morocco	73	0%	59·8 (50·0–83·0)	Urban	CLIA	32·9 (23·8)
Kazem 2014[97]	Cairo, Egypt	30	0%	48	Urban	ELISA	68·3 (9·3)
Durazo–Arvizu 2014[98]	Kumasi, Ghana<br>Victoria, Seychelles<br>Cape Town, South Africa	Ghana: 497<br>Seychelles: 494<br>South Africa: 502	50%	34 (25–45)	Ghana: rural<br>Seychelles: urban<br>South Africa: urban	LC–MS/MS	Ghana: 75·9 (6·9)<br>Seychelles: 72·9 (19·5)<br>South Africa: 59·2 (20·7)
George 2014[99,100]	Johannesburg, South Africa	Africans: 371<br>Asian–Indians: 343	48%	42 (19–65)<br>44 (18–65)	Urban	HPLC	Africans: 64·9 (46·4–89·4)<br>Asian Indians: 41·2 (28·4–56·8)
Were 2014[101]	Mombasa, Kenya	15	60%	26	Urban	CLIA	76·6 (20·5)
Aly 2014[102]	Dakahlia, Egypt	176	40%	68 (60–85)	Rural	EIA	92 (17)
El Rifai 2014[27] (mothers)	Cairo, Egypt	135	0%	26·0 (5·8)	Rural and urban	ELISA	81·4 (53·4)
Durazo–Arvizu 2013[103]	Nigeria	100	0%	31 (18–59)	Rural and urban	RIA	64 (17·4)
Hamill 2013[104]	Johannesburg, South Africa	98	0%	32 (18–49)	Urban	RIA	60 (16·5)
Luxwolda 2013[35] (adults)	Tanzania	Pregnant women: 138<br>Other adults: 60	60%	34 (16–65)	Rural	LC–MS/MS	Pregnant women: 138·5 (35·0)<br>Other adults: 115·1 (27·0)
George 2013[105]	Johannesburg, South Africa	Africans:373<br>Asians:344	48%	43 (18–70)	Urban	HPLC	Africans: 70·9 (51·5–95·1)<br>Asians: 41·8 (28·6–56·8)
Gebreegziabher 2013[106]	Rift Valley, Ethiopia	202	0%	30·8 (7·8)	Rural	ELISA	39·7 ± 9·7
El Maaty 2013[107]	Egypt	31	100%	(35–50)	Urban	HPLC	78·5 (10·5)
Olama 2013[108]	Dakahlia, Egypt	50	0%	33·1	Rural	ELISA	46·9 (13·5)
El-Shal 2013[109]	Zagazig, Egypt	150	0%	29·3	Urban	HPLC	67·9 (11·7)
Emerah 2013[110]	Zagazig, Egypt	129	0%	27·14 (20 –41)	Urban	ELISA	38·1 (15·9)
Friis 2013[111]	Mwanza, Tanzania	347	58%	NA	Urban	CLIA	84·4 (25·6)
Ibn Yacoub 2012[112]	Rabat, Morocco	30	0%	49·5	NA	na	57·41 (4·18)
Schaalan 2012[113]	Cairo, Egypt	25	72%	(30–55)	Urban	RIA	99·1 (27)
Amukele 2012[36]	Malawi	21	–	23 (22–28)	NA	LC–MS/MS	93·4 (6·5)
Luxwolda 2012[114]	Tanzania	Maasai: 35<br>Hadzabe: 25	Maasai: 43%<br>Hadzabe: 84%	34 (16–65)	Rural	LC–MS/MS	Maasai: 119·0 (26·0)<br>Hadzabe: 109·0 (28·0)
Gawad 2012[115]	Mansoura, Egypt	55	27%	38 (26–54)	Rural and urban	RIA	77·1 (16·2) pmol/L
El Maghraoui 2012[116]	Rabat, Morocco	178	0%	59 (50–79)	Urban	CLIA	46·7 (31·9
Kruger 2011[117]	Johannesburg, South Africa	658	0%	50 (35–90)	Rural and urban	CLIA	71·4 (21·9)

Nansera 2011[118]	Mbarara, Uganda	50	50%	27	NA	HPLC	64·9 (17·5)
Glew 2010[119]	Gombe, Nigeria	51	43%	52 (18–72)	Rural	LC–MS/MS	68·0 (3·7)
Allali 2009[120]	Rabat, Morocco	415	0%	50 (24–77)	Urban	RIA	45·2 (19·7)
Yan 2009[121]	Keneba, The Gambia	30	50%	67 (60–75)	Rural	RIA	68·4 (16·2)
Wejse 2007[122]	Guinea-Bissau	494	48%	37 (15–87)	Urban	LC–MS/MS	85·3 (34·8)
Alsayed 2007[123]	Cairo, Egypt	70	100%	47·1 (3·1)	Rural and urban	RIA	47·1 (3·1)
Meddeb 2005[124]	Tunis, Tunisia	261	0%	40	Urban	RIA	40
Njemini 2002[125]	Ntam, Cameroon	152	61%	66 (60–86)	Rural	RIA	52·7 (19·2)
Daniels 2000[126]	South Africa	14	NA	NA	NA	CPBA	43·4 (19·5)
Daniels 1997[127]	South Africa	139	0%	44 (20–64)	NA	CPBA	19·3 (6·8–45·6)
Sanchez 1997[49]	Maiduguri, Nigeria	Non-pregnant women: 21<br>Newborn mothers: 10	0	Non-pregnant women 17 (9·32)<br>Newborn mothers:16 (3·35)	NA	RIA	Non-pregnant women: 40·2 (9·5)<br>New mothers: 44·4 (22·8)
Garabedian 1991[128]	Constantine, Algeria	84	NA	NA	Urban	NA	Women: 27·1 (9·4)<br>Infants: 21·3 (11·8)
Feleke 1999[129]	Adis Ababa, Ethiopia	Pregnant: 31<br>Other adults: 30	0%	21 (19–40)	Urban	HPLC	Pregnant: 25 (17–46)<br>Other adults: 23·5 (18–29)
M'Buyamba-Kabangu 1987[130]	Kinshasa, Zaire	33	100%	31	Urban	RIA	64·9 (38·9)
Okonofua 1986[53] (mothers)	Ife, Nigeria	30	NA	NA	NA	CPBA	Mothers: 77·7 (15·8)
Markestad 1984[54] (mothers)	Libya	19	0%	(26–45)	NA	NA	34 (13–75)
Pettifor 1978[131]	Johannesburg, South Africa	264	NA	7·9 (1–7)	Urban	CPBA	73·9

*Data presented is only for healthy participants

[€]25(OH)D levels are presented in mean (SD) or median (IQR).

[ɜ]Vitamin D measurement assays; RIA: Radioimmunoassay; CLIA: Chemiluminescence immunoassays; LC–MS/MS: liquid chromatography-tandem mass spectrometry; HPLC–MS/MS high-performance liquid chromatography-tandem mass spectrometry; HPLC: high-performance liquid chromatography; EIA: Enzyme immunoassay; CPBA: competitive protein-binding assay. Data that were not available were indicated as NA.

Supplementary Table 5. Mean/median 25(OH)D levels for disease and control groups in case-control studies.

			Disease group		Healthy controls		
Disease	Study	Country	n	Mean (SD)/median (IQR) 25(OH)D (nmol/L)	n	Mean (SD)/median (IQR) 25(OH)D (nmol/L)	p
Rickets	Graff 2004	Nigeria	15	37·4 (13·5)	15	72·4 (4·6)	<0·0001
	Oginni 1996a	Nigeria	27	43 (5·5)	47	63 (2·6)	<0·0001
	Oginni 1996b	Nigeria	22	36 (28)	20	69 (22)	<0·0001
	Okonofua 1991	Nigeria	11	41 (22, 84)*	12	41 (29, 50)*	-
	Pfitzner 1998	Nigeria	20	56·4 (11·7)	198	64·9 (24·0)	0·12
	Walter 1997	Nigeria	16	14·1 (4·7)	27	24 (7·5)	<0·0001
	Thacher 2000	Nigeria	123	32 (22, 40)	123	50 (42, 62)*	-
	Jones 2016	Kenya	21	19 (15, 37)*	22	70 (54, 85)*	-
	Daniels 2000	South Africa	41	37·7 (15·5)	14	43·4 (19·5)	<0·0001
	Thacher 2014	Nigeria	190	34·9 (5·0, 89·9)*	257	49·9 (7·5, 127·3)*	–
Fractures	Chutterpaul 2019	South Africa	151	39·4 (23·1)	176	50·1 (23·3)	<0·0001
	El Maghraoui 2012	Morocco	97	33·5 (22)	81	46·7 (31·9)	0·001
	El Maataoui 2015	Morocco	134	39·1 (17·8)	73	32·9 (23·8)	0·035
Tuberculosis	Musarurwa 2017	Zimbabwe	134	59·9 (48·7, 73·9)	133	53·9 (45·7, 66·1) *	–
	Wejse 2007	Guinea-Bissau	362	78·3 (22·6)	494	85·3 (34·8)	<0·0001
	Ludmir 2016	Botswana	39	80·4 (53·7, 99·8)*	41	77·1 (56·4, 104·6)*	–
	Ashenafi 2018	Ethiopia	78	38·5	77	35	–
	Friis 2013	Tanzania	1223	110·9 (35·7)	347	84·4 (25·6)	<0·0001
	Boillat-Blanco 2016	Tanzania	280	94·0 (26·9)	358	89·6 (26·9)	0·041
	Edem 2016	Nigeria	24	44·08 (9·5) pg/ml	20	45·80 (13·30) pg/ml	0·62
HIV	Hamill 2013	South Africa	74	59·2 (16·5)	98	59·7 (16·5)	0·84
	Velaphi 2019 (mothers)	South Africa	137	58·8 (31·2)	152	55·5 (28·3)	0·35
	Velaphi 2019 (children)	South Africa	138	40·7 (21·2)	151	42·8 (20·9)	0·40
	Were 2014	Kenya	16	88·1 (58·2)	23	79·9 (37·4)	0·59
	Nansera 2011 (HIV only)	Uganda	50	69·9 (27·5)	50	64·9 (17·5)	0·28
	Nansera 2011 (HIV+TB)	Uganda	50	59·9 (27·5)	50	64·9 (17·5)	0·28
	Aké 2018	Ivory Coast	120	69·9 (5·5)	163	77·4 (3·9)	<0·0001
	Chikwati 2019	Zimbabwe	64	112 (33·4)	63	100 (27·1)	0·028
	Musarurwa 2017	Zimbabwe	139	50·9 (36·4, 67·1)*	133	53·9 (45·7, 66·1)*	–
Diabetes	Abd-Allah 2014	Egypt	120	35·5 (12·5)	120	46·6 (13·5)	<0·0001
	Abbiyesuku 2016	Nigeria	80	91·2 (28·2)	29	107·2 (25·2)	0·0083
	Azzam 2019	Egypt	40	35·0 (17·5)	40	40·3 (21·4)	0·23
	Bahendeka 2018	Uganda	85	116·4 (63·6)	79	99·6 (120·8)	0·26
	Azab 2013	Egypt	80	61·7 (14·0)	40	66·1 (12·0)	0·092
	Fondjo 2017	Ghana	118	6·1 (4·8, 22·3)*	100	31·3 (6·5, 81·8)*	–
	Fondjo 2017	Ghana	18	6·1 (4·8, 22·3)*	100	31·3 (6·5, 81·8)*	–
Asthma	Maalmi 2013	Tunisia	155	47·1 (16·7)	225	75·6 (14·9)	<0·0001
	Ali 2017	Egypt	82	45·2 (8·7, 136·5)*	33	90·1 (26·9, 189·1)*	–

	Lahmar 2018	Nigeria	154	48·1 (9·5)	154	70·3 (11·7)	<0·0001
	Omole 2018	Nigeria	103	122·8 (18·0)	103	127·8 (17·2)	0·043
Systemic lupus erythematosus	Abdel Galil 2018	Egypt	123	64·6 (20·5)	100	114·4 (22·9)	<0·0001
	Azab 2016	Egypt	100	84·1 (3·7)	100	53·7 (3·0)	<0·0001
	Emerah 2013	Egypt	107	27·4 (15·4)	129	38·1 (15·9)	<0·0001
	Hamza 2011	Egypt	60	65·7 (30·1)	60	106·5 (23·0)	<0·0001
Hepatitis C virus	Abdel-Mohsen 2018	Egypt	60	111·1 (21·1)	30	164·7 (7·5)	<0·0001
	Eltayeb 2015	Egypt	66	61·58 (17·05)	28	98·31 (3·50)	<0·0001
	Schaalan 2012	Egypt	50	37·2 (8·7)	25	99·1 (27·0)	<0·0001
Polycystic ovary syndrome	Bakeer 2018	Egypt	53	31·3 (14·9)	17	48·7 (27·3)	0·0013
	El-Shal 2013	Egypt	150	59·9 (13·0)	150	67·9 (11·7)	<0·0001
	Bakeer 2018	Egypt	53	31·3 (14·9)	17	48·7 (27·3)	0·0013
Cardiovascular diseases	El Maaty 2013	Egypt	63	60·1 (25·8)	31	78·5 (10·54)	<0·0001
	Derar 2017	Sudan	50	46·37 (30·74)	50	51·10 (21·91)	0·34
Vitiligo	Sobeih 2016	Egypt	75	43·7 (20·2)	75	71·9 (26·2)	<0·0001
	Ibrahim 2018	Egypt	80	34·64 (3·03)	20	76·70 (10·73)	<0·0001
Renal diseases	Cavalier 2019	Ivory Coast	100	100·3	203	64·6	–
	Derar 2017	Sudan	50	56·2 (33·6)	50	51·1 (21·9)	0·37
Fibromyalgia	Olama 2013	Egypt	50	37·7 (15·2)	50	46·9 (13·5)	0·0019
Diarrhoea	Hassam 2019	Tanzania	47	51·9	47	47·8	–
Albinism	Van der Walt 2016	South Africa	50	54·4	50	45·9	–
Alopecia areata	Fattah 2015	Egypt	30	45·9 (8·5)	30	112·8 (51·2)	<0·0001
Goiter	Aboelnaga 2016	Egypt	77	60·4 (21·7)	50	70·8 (27·2)	0·019
Graves disease	Gawad 2012	Egypt	90	53·6 (18·5)	55	77·1 (16·2)	<0·0001
Malaria	Cusick 2014	Uganda	40	52·9 (16)	20	63·1 (17·7)	0·001
Malnutrition	Nabeta 2015	Uganda	117	81·1 (30·0)	41	80·4 (27·2)	0·90
Metabolic syndrome	Alsayed 2007	Egypt	93	40 (11·3)	70	47·1 (3·1)	<0·0001
Multiple sclerosis	Skalli 2018	Morocco	113	29·2 (17·4)	146	32·4 (16·4)	0·13
Neural tube defects	Nasri 2016	Tunisia	68	20·65 (10·25)	64	28·30 (13·82)	0·0004
Pneumonia	Albanna 2010	Egypt	40	37·6 (21·1)	40	87·25 (18·4)	<0·0001
Schizophrenia	Akinlade 2017	Nigeria	60	19·8 (5·1)	30	28·1 (5·6)	<0·0001
Systemic sclerosis	Ibn Yacoub 2012	Morocco	30	27·2 (6·7)	30	57·41 (4·18)	<0·0001
β-thalassemia major	Fahim 2013	Egypt	100	10·4 (4·6)	100	40·2 (12·3)	<0·0001

*These are median values (with IQR), the rest are means (SD). Level of significance between the group means was determined using the Student t-test, this was not carried out in studies with only median 25(OH)D concentrations.

Supplementary Table 6. Seasonality in vitamin D status*

Study	Country	Seasonality in 25(OH)Dlevels	Comments
Ali 2017	Egypt	Yes	Lower vitamin D status in winter
Allali 2009	Morocco	Yes	Higher levels during summer
Ashenafi 2018	Ethiopia	No	No seasonal effects on 25(OH)Dlevels
Chikwati 2019	Zimbabwe	Yes	Summer is associated with higher vitamin D status
Djennane 2014	Algeria	Yes	Higher prevalence of vitamin D deficiency in winter
Durazo 2013	Nigeria	No	No seasonal effects on 25(OH)Dlevels
El Rifai 2014	Egypt	Yes	Higher prevalence of vitamin D deficiency in winter
Garabedian 1991	Algeria	Yes	Higher prevalence of vitamin D deficiency in winter and spring
George 2014	South Africa	Yes	Higher 25(OH)Dlevels in autumn
Poopedi 2011	South Africa	Yes	25(OH)D levels were highest in summer and autumn
Poopedi 2015	South Africa	No	No seasonal effects on 25(OH)Dlevels
Sithembiso (in press)	South Africa	Yes	25(OH)D levels were highest in summer
Sotunde 2017	South Africa	Yes	Higher 25(OH)Dlevels in autumn
Sudfeld 2017	Tanzania	Yes	Higher vitamin D status during December–March and June–August seasons
Thacher 200	Nigeria	No	No seasonal effects on 25(OH)Dlevels
Wejse 2007	Guinea-Bissau	Yes	Higher prevalence of vitamin D deficiency prevalence in winter
White 2019	South Africa	Yes	Higher prevalence of vitamin D deficiency prevalence in winter
Yan 2009	The Gambia	No	No seasonal effects on 25(OH)Dlevels

*This Table includes only studies included in this review that investigated seasonality in vitamin D status in healthy participants.

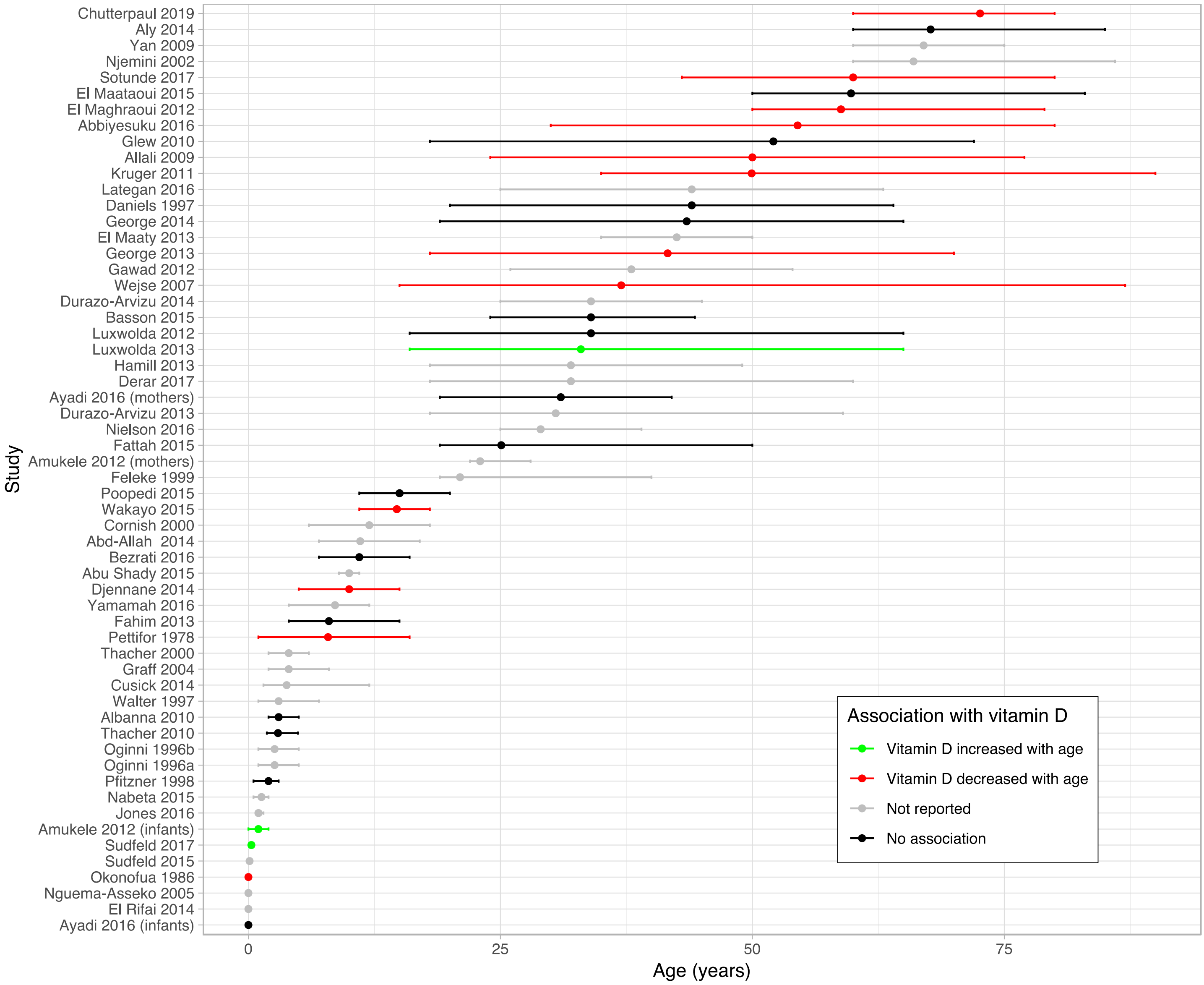

Supplementary Figure 1. Age of study participants in eligible studies and its association with 25(OH)D levels. The mean age and range are represented by the midpoints and error bars, respectively. The studies have been sorted by participants' mean age in years. Studies that had a positive association between 25(OH)D levels and age are presented in green, negative associations are presented in red, no association in black, those that did not report any association are shown in grey.

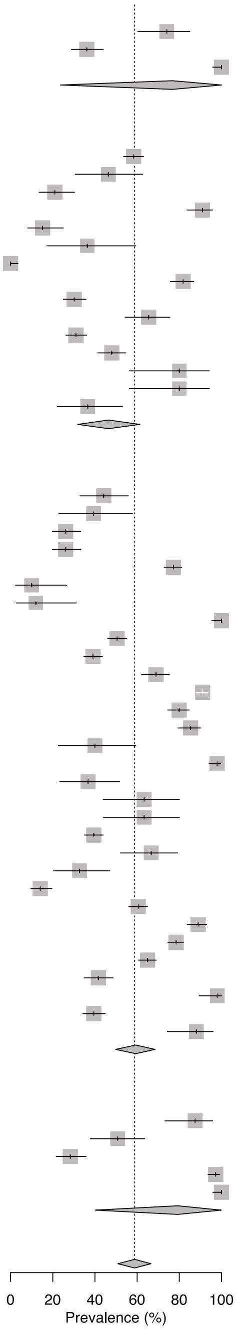

Study	Cases	n	Prevalence (%)	95% CI	Weight (%)
**A· Newborns**					
Kenya - Toko 2016	40	54	74·07	[60·35; 85·04]	1·8
Nigeria - Owie 2018	60	166	36·14	[28·84; 43·95]	1·9
Tunisia - Ayadi 2016	87	87	100·00	[95·85; 100·00]	1·9
**Random effects meta-analyis**		**307**	**76·78**	**[23·65; 100·00]**	**5·6**
Heterogeneity: I^2 = 98·86%, τ^2 = 0·24, $p < 0{\cdot}0001$					
**B· Children**					
Algeria - Djennane 2014	254	435	58·39	[53·60; 63·07]	1·9
Botwsana - Ludmir 2016	19	41	46·34	[30·66; 62·58]	1·8
Egypt - Azab 2016	21	100	21·00	[13·49; 30·29]	1·9
Egypt - Fahim 2013	91	100	91·00	[83·60; 95·80]	1·9
Egypt - Yamamah 2016	12	79	15·19	[8·10; 25·03]	1·9
Kenya - Jones 2016	8	22	36·36	[17·20; 59·34]	1·7
Nigeria - Omole 2018	0	103	0·00	[0·00; 3·52]	1·9
Nigeria - Pfitzner 1998	162	198	81·82	[75·73; 86·93]	1·9
South Africa - Poopedi 2011	89	295	30·17	[24·98; 35·76]	1·9
South Africa - White 2019	55	84	65·48	[54·31; 75·52]	1·9
Tanzania - Boillat-Blanco 2016	111	358	31·01	[26·25; 36·08]	1·9
Tunisia - Maalmi 2013	108	225	48·00	[41·31; 54·74]	1·9
Uganda - Cusick 2014	16	20	80·00	[56·34; 94·27]	1·7
Uganda - Cusick 2014	16	20	80·00	[56·34; 94·27]	1·7
Uganda - Nabeta 2015	15	41	36·59	[22·12; 53·06]	1·8
**Random effects meta-analyis**		**2121**	**46·42**	**[31·86; 61·28]**	**27·5**
Heterogeneity: I^2 = 97·72%, τ^2 = 0·080 $p < 0{\cdot}0001$					
**C· Adults (non-pregnant)**					
Egypt - Aboelnaga 2016	34	77	44·16	[32·84; 55·93]	1·9
Egypt - Ali 2017	13	33	39·39	[22·91; 57·86]	1·8
Egypt - Aly 2014	46	176	26·14	[19·81; 33·28]	1·9
Egypt - Aly 2014	46	176	26·14	[19·81; 33·28]	1·9
Egypt - Botros 2015	312	404	77·23	[72·82; 81·23]	1·9
Egypt - Fattah 2015	3	30	10·00	[2·11; 26·53]	1·8
Egypt - Schaalan 2012	3	25	12·00	[2·55; 31·22]	1·7
Ethiopia - Ashenafi 2018	78	78	100·00	[95·38; 100·00]	1·9
Ghana - Durazo -Arvizu 2014	251	497	50·50	[46·02; 54·98]	1·9
Guinea -Bissau - Wejse 2007	193	494	39·07	[34·74; 43·53]	1·9
Ivory Coast - Cavalier 2019	140	203	68·97	[62·11; 75·26]	1·9
Morocco - Allali 2009	378	415	91·08	[87·92; 93·64]	1·9
Morocco - El Maataoui 2016	203	254	79·92	[74·46; 84·67]	1·9
Morocco - El Maghraoui 2012	152	178	85·39	[79·33; 90·23]	1·9
Morocco - Ibn Yacoub 2012	12	30	40·00	[22·66; 59·40]	1·8
Morocco - Skalli 2018	143	146	97·95	[94·11; 99·57]	1·9
Nigeria - Abbiyesuku 2016	18	49	36·73	[23·42; 51·71]	1·8
Nigeria - Akinlade 2017	19	30	63·33	[43·86; 80·07]	1·8
Nigeria - Akinlade 2017	19	30	63·33	[43·86; 80·07]	1·8
Nigeria - Gbadegesin 2017	182	461	39·48	[34·99; 44·11]	1·9
Nigeria - Glew 2010	34	51	66·67	[52·08; 79·24]	1·8
Nigeria - Glew 2010	17	52	32·69	[20·33; 47·11]	1·8
Nigeria - Oluwole 2019	29	206	14·08	[9·64; 19·59]	1·9
Seychelles - Durazo -Arvizu 2014	299	494	60·53	[56·06; 64·86]	1·9
South Africa - Basson 2015	177	199	88·94	[83·74; 92·94]	1·9
South Africa - Durazo -Arvizu 2014	394	502	78·49	[74·63; 82·00]	1·9
South Africa - Myburgh 2018	328	505	64·95	[60·61; 69·11]	1·9
South Africa - Sotunde 2017	87	209	41·63	[34·87; 48·63]	1·9
South Africa - Van der Walt 2016	49	50	98·00	[89·35; 99·95]	1·8
Tanzania - Friis 2013	137	347	39·48	[34·30; 44·84]	1·9
Zimbabwe - Musarurwa 2017	37	42	88·10	[74·37; 96·02]	1·8
**Random effects meta-analyis**		**6443**	**59·35**	**[49·85; 68·51]**	**57·6**
Heterogeneity: I^2 = 98·24%, τ^2 = 0·069, $p < 0{\cdot}0001$					
**D· Pregnant women/newborn mothers**					
Egypt - Azzam 2019	35	40	87·50	[73·20; 95·81]	1·8
Kenya - Toko 2016 (mothers)	32	63	50·79	[37·89; 63·62]	1·8
Nigeria - Owie 2018 (mothers)	47	166	28·31	[21·60; 35·82]	1·9
Sudan - Gaffer 2019	175	180	97·22	[93·64; 99·09]	1·9
Tunisia - Ayadi 2016 (mothers)	87	87	100·00	[95·85; 100·00]	1·9
**Random effects meta-analyis**		**536**	**79·20**	**[40·28; 99·90]**	**9·3**
Heterogeneity: I^2 = 98·82%, τ^2 = 0·21, $p < 0{\cdot}0001$					
**Random effects meta-analyis**		**9407**	**58·83**	**[50·90; 66·54]**	**100·0**
Heterogeneity: I^2 = 98·29%, τ^2 = 0·084, $p < 0{\cdot}0001$					
Residual heterogeneity: I^2 = 98·24%, $p < 0{\cdot}0001$					
Test for subgroup differences: χ^2_3 = 4·07, df = 3 (p = 0·25)					

Supplementary Figure 2. Pooled prevalence of low vitamin D status in the general population in Africa using <75 nmol/L cut-off. Cases were defined as number of participants in a study with 25(OH)D levels <75 nmol/L and n as the total number of participants in the study.

A. 75 nmol/L cutoff

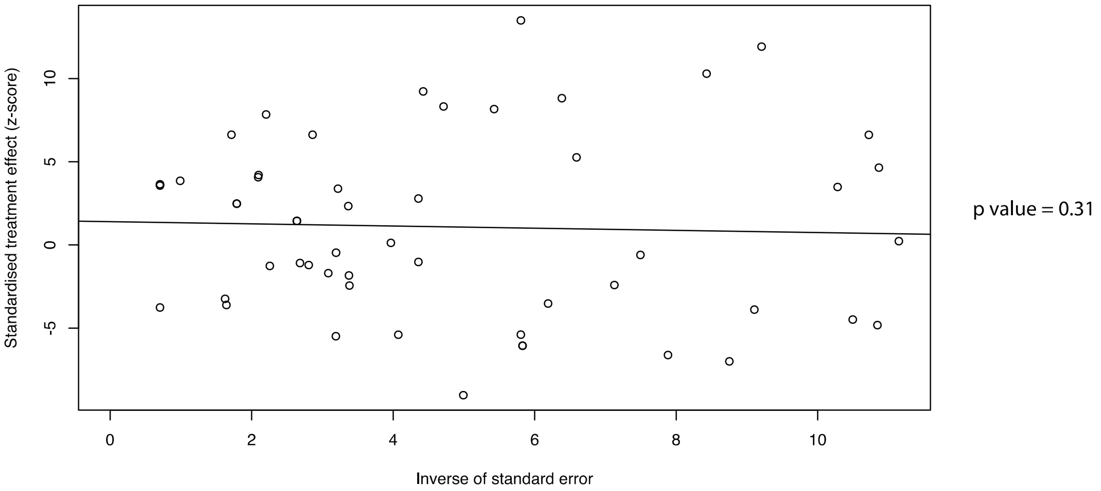

B. 50 nmol/L cutoff

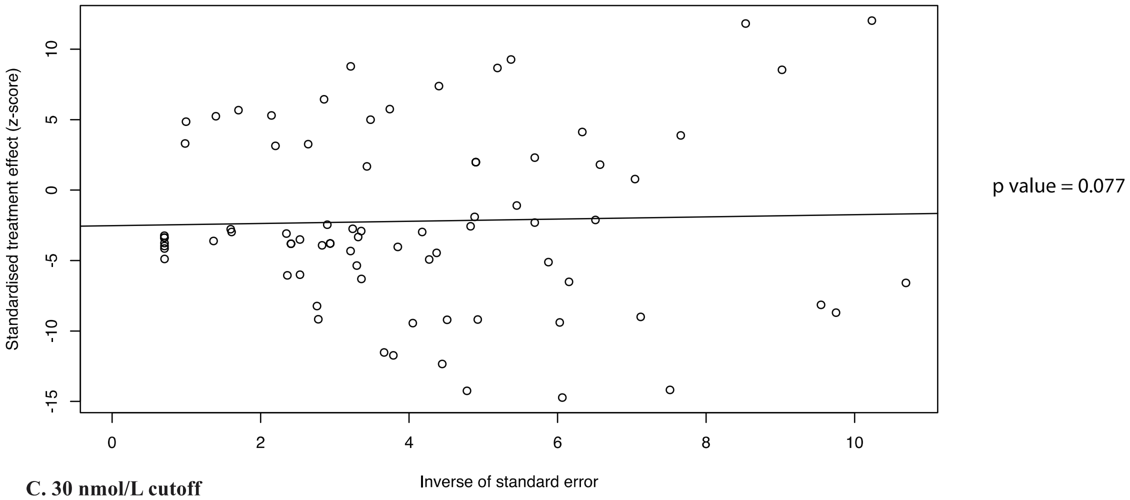

C. 30 nmol/L cutoff

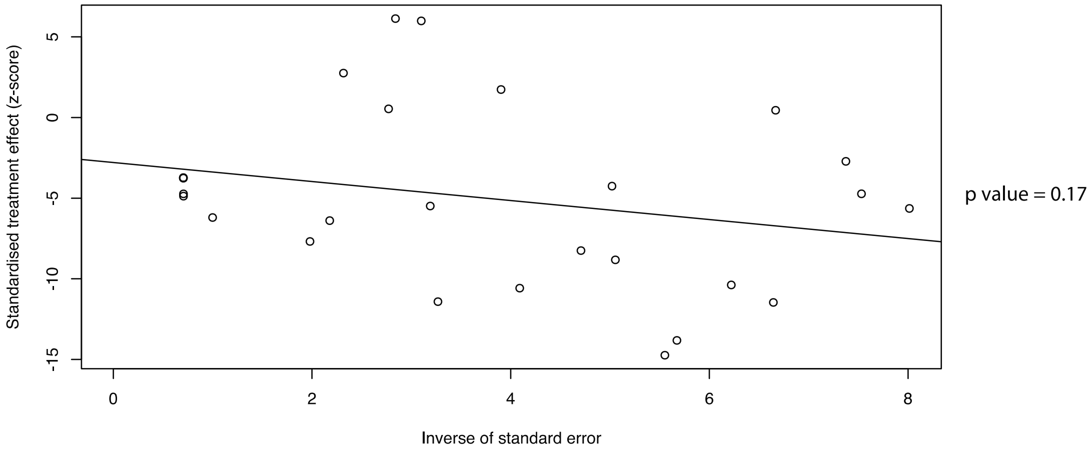

Supplementary Figure 3. Funnel plot asymmetry tests. The asymmetry of the funnel plots for the meta-analyses of prevalence was tested using Egger test of bias[133], where a linear regression method was used. P <0·05 indicated significant publication bias.

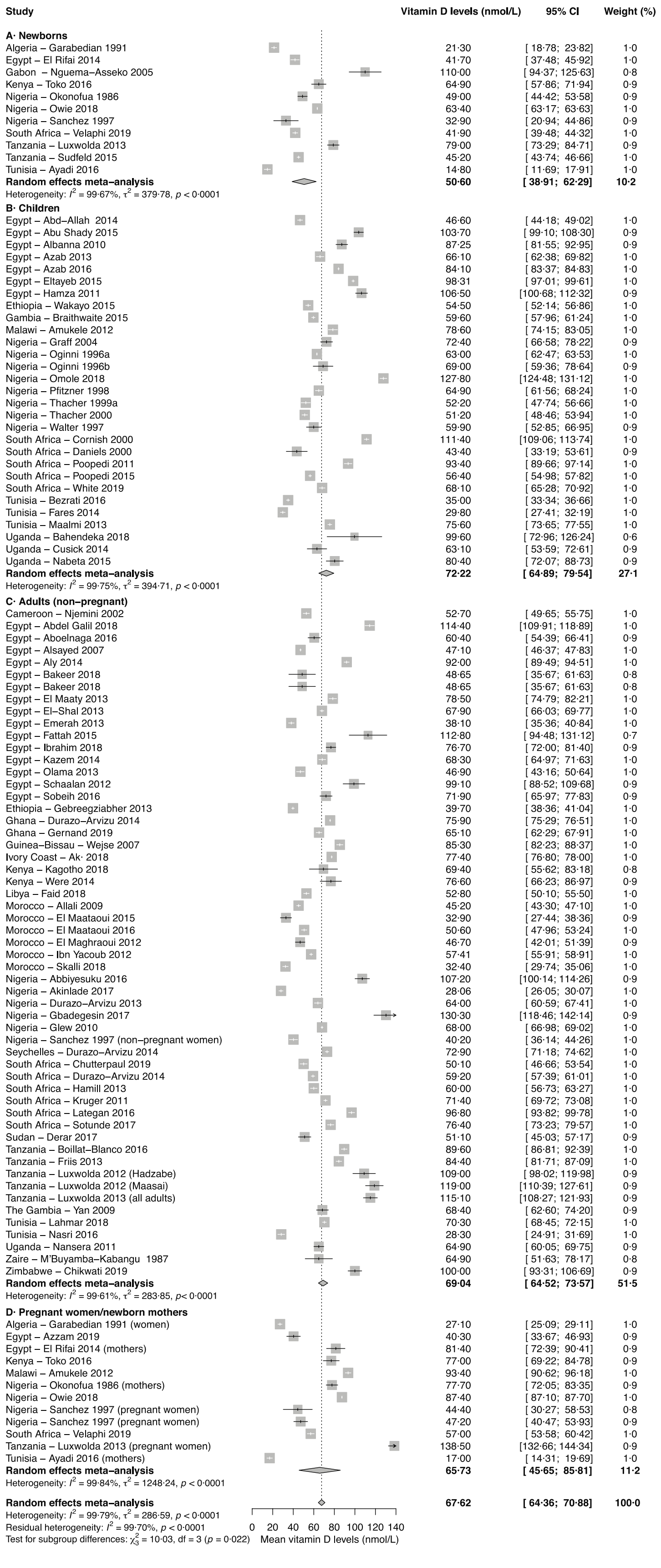

Supplementary Figure 4. Pooled mean 25(OH)D levels in the general population in Africa stratified by age group. Studies that only reported median 25(OH)D values were excluded from meta-analysis.

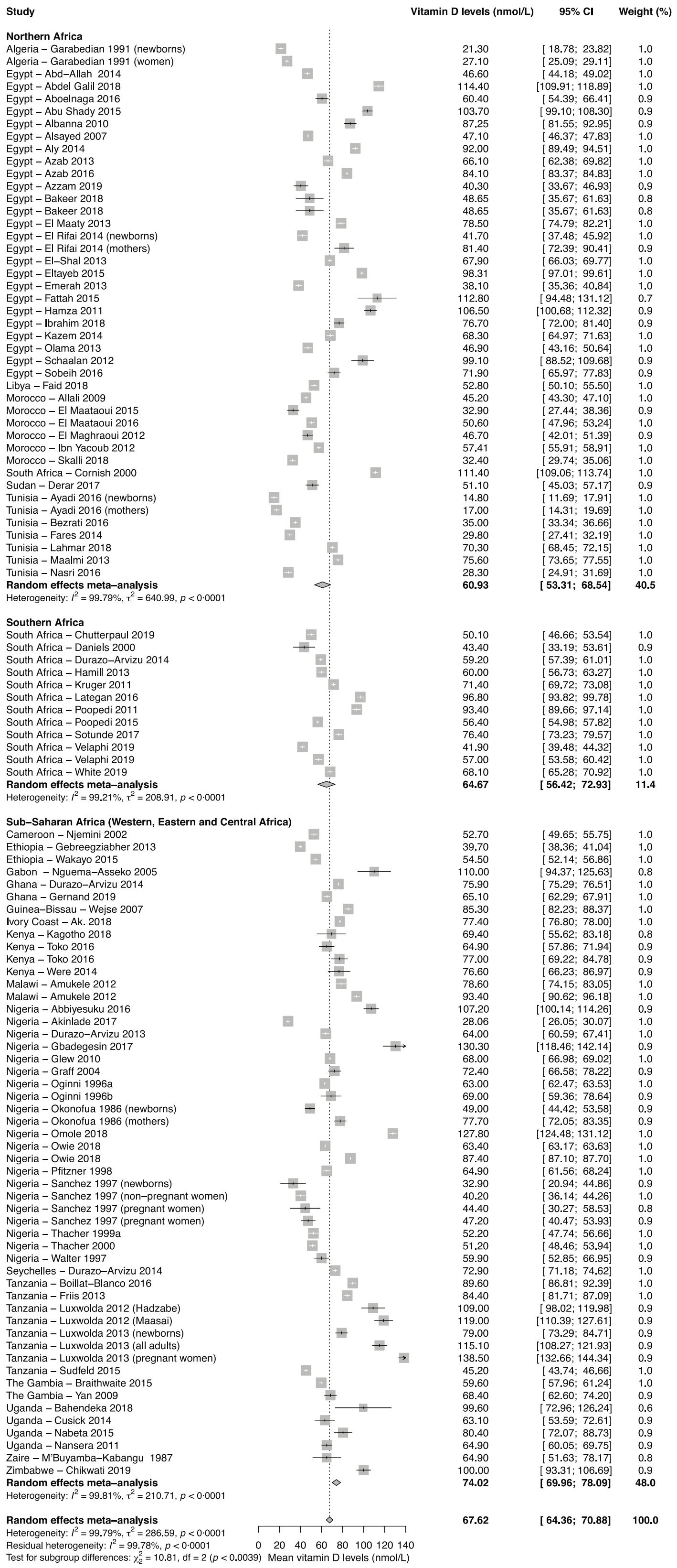

Supplementary Figure 5. 25(OH)D levels stratified by UN African Regions. Northern African countries and South Africa were compared with sub-Saharan Africa including West, East and Central African regions.

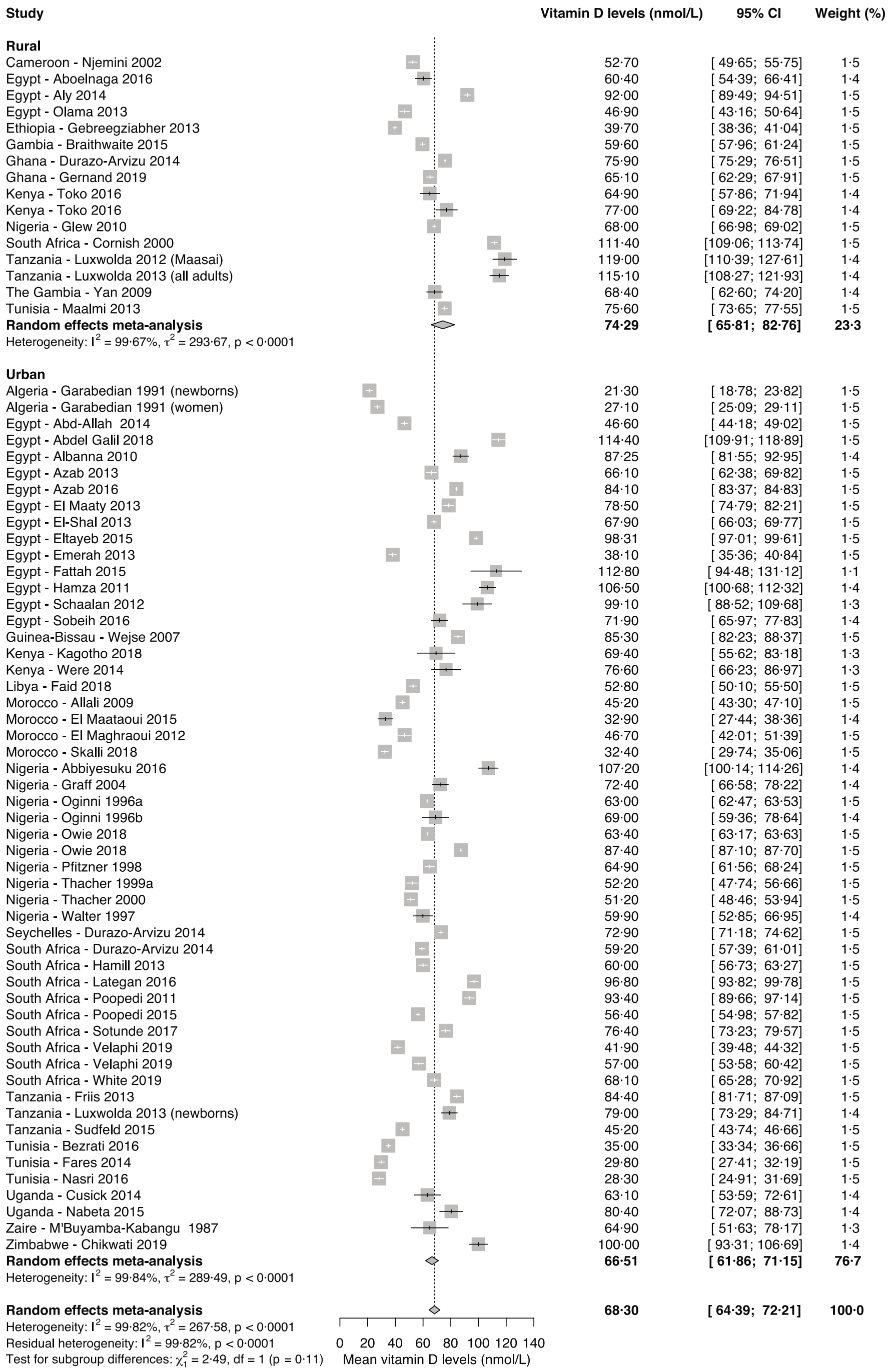

Supplementary Figure 6. 25(OH)D levels stratified by area (rural vs urban)

A· Mean differences in 25(OH)D levels between men and women from the same study

Study	Men Total	Men Mean	Men SD	Women Total	Women Mean	Women SD	MD in 25(OH)D (nmol/L)	95% CI	Weight (%)
Egypt - Abu Shady 2015	98	98·50	30·90	102	108·80	35·40	-10·30	[-19·50; -1·10]	12·1
Ethiopia - Wakayo 2015	75	60·30	16·90	99	50·00	13·50	10·30	[5·64; 14·96]	13·9
Kenya - Kagotho 2018	191	70·50	111·00	62	62·60	85·20	7·90	[-18·51; 34·31]	5·4
Libya - Faid 2018	75	52·80	30·00	380	52·80	29·30	0·00	[-7·40; 7·40]	12·9
Nigeria - Glew 2010	22	79·60	4·00	29	59·20	3·50	20·40	[18·30; 22·50]	14·5
South Africa - Lategan 2016	76	108·60	29·45	263	92·35	26·46	16·25	[8·90; 23·60]	12·9
South Africa - Poopedi 2011	155	100·00	34·40	144	86·00	31·10	14·00	[6·58; 21·42]	12·9
Tanzania - Luxwolda 2013 (Hadzabe)	21	106·30	29·20	4	123·80	18·00	-17·50	[-39·11; 4·11]	6·8
Tanzania - Luxwolda 2013 (Maasai)	15	120·90	24·00	20	118·30	27·40	2·60	[-14·48; 19·68]	8·5
**Random effects meta-analysis**	**728**			**1103**			**6·51**	**[-1·19; 14·20]**	**100·0**

Heterogeneity: $I^2 = 90\%$, $\tau^2 = 105·11$, $p < 0·0001$

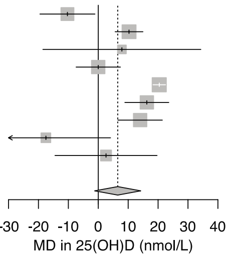

B· Mean differences in 25(OH)D levels between mothers and their newborns from the same study

Study	Mothers Total	Mothers Mean	Mothers SD	Newborns Total	Newborns Mean	Newborns SD	MD in 25(OH)D (nmol/L)	95% CI	Weight (%)
Algeria - Garabedian 1991	84	27·10	9·40	84	21·30	11·80	5·80	[2·57; 9·03]	11·3
Egypt - El Rifai 2014	135	81·40	53·40	135	41·70	25·00	39·70	[29·75; 49·65]	9·6
Kenya - Toko 2016	63	77·00	31·50	54	64·90	26·40	12·10	[1·61; 22·59]	9·5
Malawi - Amukele 2012	21	93·40	6·50	21	78·60	10·40	14·80	[9·55; 20·05]	10·9
Nigeria - Okonofua 1986	30	77·70	15·80	30	49·00	12·80	28·70	[21·42; 35·98]	10·4
Nigeria - Owie 2018	166	87·40	2·00	166	63·40	1·50	24·00	[23·62; 24·38]	11·5
Nigeria - Sanchez 1997	10	44·40	22·80	10	32·90	19·30	11·50	[-7·01; 30·01]	6·8
South Africa - Velaphi 2019	290	57·00	29·70	290	41·90	21·00	15·10	[10·91; 19·29]	11·2
Tanzania - Luxwolda 2013	25	135·90	32·00	25	90·60	28·20	45·30	[28·58; 62·02]	7·4
Tunisia - Ayadi 2016	87	17·00	12·80	87	14·80	10·40	2·20	[-1·27; 5·67]	11·3
**Random effects meta-analysis**	**911**			**902**			**19·08**	**[11·49; 26·68]**	**100·0**

Heterogeneity: $I^2 = 97·17\%$, $\tau^2 = 130·23$, $p < 0·0001$

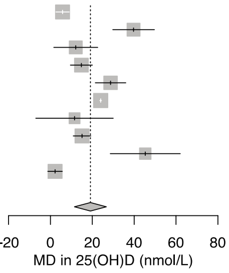

C· Mean differences in 25(OH)D levels between rural and urban inhabitants from the same study

Study	Rural Total	Rural Mean	Rural SD	Urban Total	Urban Mean	Urban SD	MD in 25(OH)D (nmol/L)	95% CI	Weight (%)
Ethiopia - Wakayo 2015	85	61·00	15·10	89	48·20	14·00	12·80	[8·47; 17·13]	33·1
Nigeria - Durazo-Arvizu 2013	100	64·40	17·40	95	29·10	12·70	35·30	[31·04; 39·56]	33·1
South Africa - Kruger 2011	324	76·40	10·03	324	68·17	5·27	8·23	[7·00; 9·46]	33·8
**Random effects meta-analysis**	**509**			**508**			**18·70**	**[2·92; 34·48]**	**100·0**

Heterogeneity: $I^2 = 99\%$, $\tau^2 = 191·13$, $p < 0·0001$

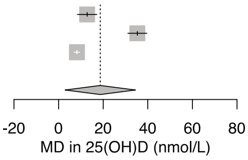

D· Mean differences in 25(OH)D levels between rickets cases and healthy controls from the same study

Study	Rickets cases Total	Rickets cases Mean	Rickets cases SD	Healthy controls Total	Healthy controls Mean	Healthy controls SD	MD in 25(OH)D (nmol/L)	95% CI	Weight (%)
Nigeria - Graff 2004	15	37·40	13·50	15	72·40	4·60	-35·00	[-42·22; -27·78]	17·2
Nigeria - Oginni 1996a	27	43·00	5·50	47	63·00	2·60	-20·00	[-22·20; -17·80]	20·0
Nigeria - Oginni 1996b	22	36·00	28·00	20	69·00	22·00	-33·00	[-48·16; -17·84]	11·3
Nigeria - Pfitzner 1998	20	56·40	11·70	198	64·90	24·00	-8·50	[-14·62; -2·38]	18·0
Nigeria - Walter 1997	16	14·10	4·70	27	24·00	7·50	-9·90	[-13·55; -6·25]	19·4
South Africa - Daniels 2000	41	37·70	15·50	14	43·40	19·50	-5·70	[-16·96; 5·56]	14·1
**Random effects meta-analysis**	**141**			**321**			**-18·00**	**[-25·64; -10·36]**	**100·0**

Heterogeneity: $I^2 = 92\%$, $\tau^2 = 74·75$, $p < 0·0001$

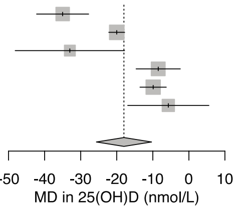

Supplementary Figure 7. Mean difference (MD) in 25(OH)D levels between men and women (A), between mothers and their infants (B), between rural and urban inhabitants (C), and between rickets cases and healthy controls (D). These were differences in mean 25(OH)D levels between the groups in the same study.

References

1. Sithembiso C. Velaphi, Shabir A. Madhi, Alane Izu, et al. Maternal and neonatal vitamin D status at birth in black South Africans. *S Afr Med J* (in press).
2. Hassam I, Kisenge R, Aboud S, et al. Association of vitamin D and diarrhoea in children aged less than five years at Muhimbili national hospital, Dar es Salaam: An unmatched case control study. *BMC Pediatr* 2019; **19**(1): 237.
3. Houghton LA, Brown RC, Beaumont S, et al. Micronutrient status differs among Maasai and Kamba preschoolers in a supplementary feeding programme in Kenya. *Maternal and Child Nutrition* 2019; **15**(3): e12805.
4. Bodin J, Mihret A, Holm-Hansen C, et al. Vitamin D Deficiency is Associated with Increased Use of Antimicrobials among Preschool Girls in Ethiopia. *Nutrients* 2019; **11**(3).
5. White Z, White S, Dalvie T, et al. Bone health, body composition, and vitamin D status of black preadolescent children in South Africa. *Nutrients* 2019; **11**(6): 1243.
6. Owie E, Afolabi BB. Vitamin D deficiency in pregnant women and newborns in Lagos, Nigeria. *J Obstet Gynaecol* 2018; **38**(5): 616-21.
7. Youssef MAM, Zahran AM, Hussien AM, et al. In neonates with vitamin D deficiency, low lymphocyte activation markers are risk factors for infection. *Paediatrics and International Child Health* 2019; **39**(2): 111-8.
8. Omole KO, Kuti BP, Oyelami OA, et al. Serum vitamin D profile of Nigerian children with asthma: Association with asthma severity and control. *Pediatr Pulmonol* 2018; **53**(5): 544-51.
9. Sudfeld CR, Manji KP, Smith ER, et al. Vitamin D Deficiency Is Not Associated With Growth or the Incidence of Common Morbidities Among Tanzanian Infants. *J Pediatr Gastroenterol Nutr* 2017; **65**(4): 467-74.
10. Ayadi ID, Nouaili EB, Talbi E, et al. Prevalence of vitamin D deficiency in mothers and their newborns in a Tunisian population. *Int J Gynaecol Obstet* 2016; **133**(2): 192-5.
11. Jones KDJ, Hachmeister CU, Khasira M, et al. Vitamin D deficiency causes rickets in an urban informal settlement in Kenya and is associated with malnutrition. *Matern Child Nutr* 2018; **14**(1).
12. Bezrati I, Ben Fradj MK, Ouerghi N, et al. Vitamin D inadequacy is widespread in Tunisian active boys and is related to diet but not to adiposity or insulin resistance. *Libyan J Med* 2016; **11**: 31258.
13. Yamamah GA, Abdelhamid EN, Farid TM, et al. Simple screening procedures for evaluation of some health problems in children living at remote areas. *Research Journal of Pharmaceutical, Biological and Chemical Sciences* 2016; **7**(4): 1750-5.
14. Toko EN, Sumba OP, Daud, II, et al. Maternal Vitamin D Status and Adverse Birth Outcomes in Children from Rural Western Kenya. *Nutrients* 2016; **8**(12).
15. Boillat-Blanco N, Bovet P, Ramaiya KL, et al. Association between tuberculosis, diabetes and 25 hydroxyvitamin D in Tanzania: a longitudinal case control study. *BMC Infect Dis* 2016; **16**(1): 626.
16. Ludmir J, Mazhani L, Cary MS, et al. Vitamin D Status in Botswana Children Under 2 Years Old With and Without Active Tuberculosis. *Am J Trop Med Hyg* 2016; **94**(5): 971-4.
17. Eltayeb AA, Abdou MA, Abdel-aal AM, et al. Vitamin D status and viral response to therapy in hepatitis C infected children. *World J Gastroenterol* 2015; **21**(4): 1284-91.
18. Braithwaite VS, Jones KS, Schoenmakers I, et al. Vitamin D binding protein genotype is associated with plasma 25OHD concentration in West African children. *Bone* 2015; **74**: 166-70.
19. Sudfeld CR, Duggan C, Aboud S, et al. Vitamin D status is associated with mortality, morbidity, and growth failure among a prospective cohort of HIV-infected and HIV-exposed Tanzanian infants. *J Nutr* 2015; **145**(1): 121-7.

20. Poopedi MA, Norris SA, Micklesfield LK, et al. Does vitamin D status track through adolescence? *Am J Clin Nutr* 2015; **102**(5): 1025-9.
21. Wakayo T, Belachew T, Vatanparast H, et al. Vitamin D deficiency and its predictors in a country with thirteen months of sunshine: the case of school children in central Ethiopia. *PLoS One* 2015; **10**(3): e0120963.
22. Wakayo T, Belachew T, Whiting SJ. Serum Vitamin D Level Associates With Handgrip Muscle Strength Among Ethiopian Schoolchildren: A Cross-Sectional Study. *Food Nutr Bull* 2018; **39**(1): 54-64.
23. Shady MM, Youssef MM, Shehata MA, et al. Association of Serum 25-Hydroxyvitamin D with Life Style and Dietary Factors in Egyptian Prepubescent Children. *Open Access Maced J Med Sci* 2015; **3**(1): 80-4.
24. Nabeta HW, Kasolo J, Kiggundu RK, et al. Serum vitamin D status in children with protein-energy malnutrition admitted to a national referral hospital in Uganda. *BMC Res Notes* 2015; **8**: 418.
25. Cusick SE, Opoka RO, Lund TC, et al. Vitamin D insufficiency is common in Ugandan children and is associated with severe malaria. *PLoS One* 2014; **9**(12): e113185.
26. Djennane M, Lebbah S, Roux C, et al. Vitamin D status of schoolchildren in Northern Algeria, seasonal variations and determinants of vitamin D deficiency. *Osteoporos Int* 2014; **25**(5): 1493-502.
27. El Rifai NM, Abdel Moety GA, Gaafar HM, et al. Vitamin D deficiency in Egyptian mothers and their neonates and possible related factors. *J Matern Fetal Neonatal Med* 2014; **27**(10): 1064-8.
28. El Sakka AS, Imam SS, Amer HA, et al. Vitamin D deficiency and low hemoglobin level as risk factors for severity of acute lower respiratory tract infections in Egyptian children: A case-control study. *Egyptian Pediatric Association Gazette* 2014; **62**(1): 1-7.
29. Abd-Allah SH, Pasha HF, Hagrass HA, et al. Vitamin D status and vitamin D receptor gene polymorphisms and susceptibility to type 1 diabetes in Egyptian children. *Gene* 2014; **536**(2): 430-4.
30. Fares S, Sethom MM, Khouaja-Mokrani C, et al. VitaminA, E, and D deficiencies in tunisian very low birth weight neonates: prevalence and risk factors. *Pediatr Neonatol* 2014; **55**(3): 196-201.
31. Thacher TD, Fischer PR, Pettifor JM. The effect of nutritional rickets on bone mineral density. *J Clin Endocrinol Metab* 2014; **99**(11): 4174-80.
32. Maalmi H, Sassi FH, Berraies A, et al. Association of vitamin D receptor gene polymorphisms with susceptibility to asthma in Tunisian children: A case control study. *Hum Immunol* 2013; **74**(2): 234-40.
33. Azab SF, Saleh SH, Elsaeed WF, et al. Vitamin D status in diabetic Egyptian children and adolescents: a case-control study. *Ital J Pediatr* 2013; **39**: 73.
34. Fahim FM, Saad K, Askar EA, et al. Growth Parameters and Vitamin D status in Children with Thalassemia Major in Upper Egypt. *Int J Hematol Oncol Stem Cell Res* 2013; **7**(4): 10-4.
35. Luxwolda MF, Kuipers RS, Kema IP, et al. Vitamin D status indicators in indigenous populations in East Africa. *Eur J Nutr* 2013; **52**(3): 1115-25.
36. Amukele TK, Soko D, Katundu P, et al. Vitamin D levels in Malawian infants from birth to 24 months. *Arch Dis Child* 2013; **98**(3): 180-3.
37. Hamza RT, Awwad KS, Ali MK, et al. Reduced serum concentrations of 25-hydroxy vitamin D in Egyptian patients with systemic lupus erythematosus: relation to disease activity. *Med Sci Monit* 2011; **17**(12): Cr711-8.
38. Poopedi MA, Norris SA, Pettifor JM. Factors influencing the vitamin D status of 10-year-old urban South African children. *Public Health Nutr* 2011; **14**(2): 334--9.

39. Thacher TD, Fischer PR, Obadofin MO, et al. Comparison of metabolism of vitamins D2 and D3 in children with nutritional rickets. *J Bone Miner Res* 2010; **25**(9): 1988-95.
40. Albanna EAM, Ali YF, Reayid AM. Vitamin D and LL-37 in children with pneumonia. *Egypt J Pediatr Allergy Immunol* 2010; **8**(2): 81--6.
41. Nguema-Asseko B, Ganga-Zandzou PS, Ovono F, et al. [Vitamin D status in Gabonese children]. *Archives de pediatrie : organe officiel de la Societe francaise de pediatrie* 2005; **12**(11): 1587-90.
42. Graff M, Thacher TD, Fischer PR, et al. Calcium absorption in Nigerian children with rickets. *Am J Clin Nutr* 2004; **80**(5): 1415-21.
43. Thacher TD, Fischer PR, Pettifor JM, et al. Case-control study of factors associated with nutritional rickets in Nigerian children. *J Pediatr* 2000; **137**(3): 367-73.
44. Thacher TD, Fischer PR, Pettifor JM, et al. A comparison of calcium, vitamin D, or both for nutritional rickets in Nigerian children. *N Engl J Med* 1999; **341**(8): 563-8.
45. Thacher T, Glew RH, Isichei C, et al. Rickets in Nigerian children: response to calcium supplementation. *J Trop Pediatr* 1999; **45**(4): 202-7.
46. Pfitzner MA, Thacher TD, Pettifor JM, et al. Absence of vitamin D deficiency in young Nigerian children. *J Pediatr* 1998; **133**(6): 740-4.
47. Cornish DA, Maluleke V, Mhlanga T. An investigation into a possible relationship between vitamin D, parathyroid hormone, calcium and magnesium in a normally pigmented and an albino rural black population in the Northern Province of South Africa. *Biofactors* 2000; **11**(1-2): 35-8.
48. Walter EA, Scariano JK, Easington CR, et al. Rickets and protein malnutrition in northern Nigeria. *J Trop Pediatr* 1997; **43**(2): 98-102.
49. Sanchez PA, Idrisa A, Bobzom DN, et al. Calcium and vitamin D status of pregnant teenagers in Maiduguri, Nigeria. *J Natl Med Assoc* 1997; **89**(12): 805-11.
50. Oginni LM, Worsfold M, Sharp CA, et al. Plasma osteocalcin in healthy Nigerian children and in children with calcium-deficiency rickets. *Calcif Tissue Int* 1996; **59**(6): 424-7.
51. Oginni LM, Worsfold M, Oyelami OA, et al. Etiology of rickets in Nigerian children. *J Pediatr* 1996; **128**(5 Pt 1): 692-4.
52. Okonofua F, Gill DS, Alabi ZO, et al. Rickets in Nigerian children: a consequence of calcium malnutrition. *Metabolism* 1991; **40**(2): 209-13.
53. Okonofua F, Houlder S, Bell J, et al. Vitamin D nutrition in pregnant Nigerian women at term and their newborn infants. *J Clin Pathol* 1986; **39**(6): 650-3.
54. Markestad T, Elzouki A, Legnain M, et al. Serum concentrations of vitamin D metabolites in maternal and umbilical cord blood of Libyan and Norwegian women. *Hum Nutr Clin Nutr* 1984; **38**(1): 55-62.
55. Oluwole AA, Okunade KS, Okojie OE. Maternal serum vitamin D levels and preterm delivery among low-risk parturients in Lagos, Nigeria. *Int J Gynaecol Obstet* 2019; **144**(2): 216-20.
56. Azzam EZ, El-Aghoury AA, Abd El-naby ESE, et al. Studying the relation between vitamin D deficiency and glycemic state among pregnant women with gestational diabetes. *Diabetes and Metabolic Syndrome: Clinical Research and Reviews* 2019; **13**(2): 1505-9.
57. Cavalier E, Sagou Yayo E, Attoungbre-Hauhouot ML, et al. Vitamin D, bone alkaline phosphatase and parathyroid hormone in healthy subjects and haemodialysed patients from West Africa: impact of reference ranges and parathyroid hormone generation assays on the KDIGO guidelines. *Clinical Kidney Journal* 2018; **12**(2): 288-93.
58. Chikwati RP, Musarurwa C, Duri K, et al. Maternal plasma vitamin D levels and associated determinants in late pregnancy in Harare, Zimbabwe: A cross-sectional study. *BMC Pregnancy Childbirth* 2019; **19**(1): 218.

59. Chutterpaul P, Paruk F, Cassim B. Prevalence of Vitamin D deficiency in older South Africans with and without hip fractures and the effects of age, body weight, ethnicity and functional status. *Journal of Endocrinology, Metabolism and Diabetes of South Africa* 2019; **24**(1): 10-5.
60. Gaffer AA, Rayis DA, Elhussein OG, et al. Vitamin D status in Sudanese pregnant women: a cross-sectional study. *Trans R Soc Trop Med Hyg* 2019.
61. Gernand AD, Aguree S, Pobee R, et al. Concurrent Micronutrient Deficiencies Are Low and Micronutrient Status Is Not Related to Common Health Indicators in Ghanaian Women Expecting to Become Pregnant. *Current Developments in Nutrition* 2019; **3**(6): nzz053.
62. Younouss AAMB, Moor VJA, Bidjoni LB, et al. Vitamin D deficiency in pregnant women attending prenatal consultations in Yaounde (Cameroon). *Clin Chim Acta* 2019; **493**(Supplement 1): S619.
63. Myburgh PH, Towers GW, Kruger IM, et al. CRP Genotypes Predict Increased Risk to Co-Present with Low Vitamin D and Elevated CRP in a Group of Healthy Black South African Women. *Int J Environ Res Public Health* 2018; **15**(1).
64. Kagotho E, Omuse G, Okinda N, et al. Vitamin D status in healthy black African adults at a tertiary hospital in Nairobi, Kenya: a cross sectional study. *BMC Endocr Disord* 2018; **18**(1): 70.
65. Faid F, Nikolic M, Milesevic J, et al. Assessment of vitamin D intake among Libyan women - adaptation and validation of specific food frequency questionnaire. *Libyan J Med* 2018; **13**(1): 1502028.
66. Ibrahim H, El Taieb M, El Gamel Z, et al. Effect of narrow-band ultraviolet B on the serum of 25-hydroxyvitamin D in vitiligo patients. *J Cosmet Dermatol* 2018; **17**(5): 911-6.
67. Ashenafi S, Mazurek J, Rehn A, et al. Vitamin D(3) Status and the Association with Human Cathelicidin Expression in Patients with Different Clinical Forms of Active Tuberculosis. *Nutrients* 2018; **10**(6).
68. Skalli A, Ait Ben Haddou EH, El Jaoudi R, et al. Association of vitamin D status with multiple sclerosis in a case-control study from Morocco. *Rev Neurol (Paris)* 2018; **174**(3): 150-6.
69. Lahmar O, Salhi M, Kaabachi W, et al. Association Between Vitamin D Metabolism Gene Polymorphisms and Risk of Tunisian Adults' Asthma. *Lung* 2018; **196**(3): 285-95.
70. Aké AJA, Boyvin L, Séri KL, et al. Metabolic Disorders of Vitamin D and Parathyroid Hormone in Patients Living with Human Immunodeficiency Virus Type 1 Taking Antiretroviral Treatment in Côte d'Ivoire. *International STD Research & Reviews* 2018; **7**(2): 1-10.
71. Bahendeka S, Wesonga R, Were TP, et al. Autoantibodies and HLA class II DR-DQ genotypes in Ugandan children and adolescents with type 1 diabetes mellitus. *Int J Diabetes Dev Ctries* 2019; **39**(1): 39-46.
72. Bakeer E, Radwan R, El Mandoury A, et al. Anti-Müllerian Hormone as a Diagnostic Marker in Egyptian Infertile Polycystic Ovary Syndrome Females: Correlations with Vitamin D, Total Testosterone, Dyslipidemia and Anthropometric Parameters. *Journal of Medical Biochemistry* 2018; **37**(4): 448-55.
73. Fondjo LA, Owiredu W, Sakyi SA, et al. Vitamin D status and its association with insulin resistance among type 2 diabetics: A case -control study in Ghana. *PLoS One* 2017; **12**(4): e0175388.
74. Ali AM, Selim S, Abbassi MM, et al. Effect of alfacalcidol on the pulmonary function of adult asthmatic patients: A randomized trial. *Ann Allergy Asthma Immunol* 2017; **118**(5): 557-63.

75. Musa IR, Rayis DA, Ahmed MA, et al. Thyroid Function and 25 (OH) Vitamin D Level among Sudanese Women in Early Pregnancy. *Open Access Macedonian Journal of Medical Sciences* 2018; **6**(3): 488-92.
76. Abdel Galil SM, El-Shafey AM, Abdul-Maksoud RS, et al. Interferon alpha gene expression and serum level association with low vitamin D levels in Egyptian female patients with systemic lupus erythematosus. *Lupus* 2018; **27**(2): 199-209.
77. Sotunde OF, Kruger HS, Wright HH, et al. Association of 25-hydroxyvitamin D and parathyroid hormone with the metabolic syndrome in black South African women. *Appl Physiol Nutr Metab* 2017; **42**(4): 413-9.
78. Akinlade KS, Olaniyan OA, Lasebikan VO, et al. Vitamin D Levels in Different Severity Groups of Schizophrenia. *Front Psychiatry* 2017; **8**(JUN): 105.
79. Gbadegesin A, Sobande A, Adedeji O, et al. Maternal serum vitamin D levels and pregnancy outcomes: from Lagos, Nigeria. *J Obstet Gynaecol* 2017; **37**(1): 25-8.
80. Derar TMO, Gader AGMA, Kordofani AAY, et al. Role of cystathionine beta synthase gene variant in sudanese population. *Biomedical Research (India)* 2017; **28**(13): 6059-63.
81. Musarurwa C, Zijenah LS, Duri DZ, et al. Association of high serum vitamin D concentrations with active pulmonary TB in an HIV co-endemic setting, Harare, Zimbabwe. *BMC Infect Dis* 2017; **17**(1): 142.
82. Nielson CM, Jones KS, Chun RF, et al. Free 25-Hydroxyvitamin D: Impact of Vitamin D Binding Protein Assays on Racial-Genotypic Associations. *The Journal of Clinical Endocrinology & Metabolism* 2016; **101**(5): 2226-34.
83. Lategan R, Van den Berg VL, Ilich JZ, et al. Vitamin D status, hypertension and body mass index in an urban black community in Mangaung, South Africa. *Afr J Prim Health Care Fam Med* 2016; **8**(1): e1-e5.
84. El Maataoui A, Biaz A, El Machtani S, et al. Vitamin D status in healthy Moroccan men and women aged 50 years and older: a cross-sectional study. *Arch Osteoporos* 2016; **11**(1): 24.
85. Edem VF, Ige O, Arinola OG. Plasma vitamins and essential trace elements in multi-drug resistant tuberculosis patients before and during chemotherapy. *Egyptian Journal of Chest Diseases and Tuberculosis* 2016; **65**(2): 441-5.
86. Abbiyesuku F, Olawale O, Agbakwuru A, et al. Vitamin D Levels and Insulin Resistance among Nigerian Men with Type-2 Diabetes mellitus. *Annals of Health Research* 2016; **2**(1): 10-6.
87. Aboelnaga MM, Elshafei MM, Elsayed E. Vitamin D status in Egyptian euthyroid multinodular non-toxic goiter patients and its correlation with TSH levels. *Endocrinol Nutr* 2016; **63**(8): 380-6.
88. Van der Walt JE, Sinclair W. Vitamin D levels in patients with albinism compared with those in normally pigmented Black patients attending dermatology clinics in the Free State province, South Africa. *Int J Dermatol* 2016; **55**(9): 1014-9.
89. Azab SF, Ali YF, Farghaly MA, et al. Vitamin D receptor gene BsmI polymorphisms in Egyptian children and adolescents with systemic lupus erythematosus: A case-control study. *Medicine (Baltimore)* 2016; **95**(46): e5233.
90. Ben Fradj MK, Gargouri MM, Hammami MB, et al. Bladder Cancer is Associated with Low Plasma 25-hydroxyvitamin D Concentrations in Tunisian Population. *Nutr Cancer* 2016; **68**(2): 208-13.
91. Nasri K, Ben Fradj MK, Feki M, et al. Maternal 25-hydroxyvitamin D level and the occurrence of neural tube defects in Tunisia. *Int J Gynaecol Obstet* 2016; **134**(2): 131-4.

92. Sobeih S, Mashaly HM, Gawdat H, et al. Evaluation of the correlation between serum levels of vitamin D and vitamin D receptor gene polymorphisms in an Egyptian population. *Int J Dermatol* 2016; **55**(12): 1329-35.
93. Basson A, Swart R, Jordaan E, et al. Vitamin D deficiency increases the risk for moderate to severe disease activity in Crohn's disease patients in South Africa, measured by the Harvey Bradshaw Index. *South African Gastroenterology Review* 2015; **13**(2): 50.
94. Abdel Fattah NSA, Darwish YW. Assessment of serum 25-hydroxyvitamin D levels in patients with extensive/recalcitrant alopecia areata before and after PUVA and NB-UVB therapy. *Journal of the Egyptian Women's Dermatologic Society* 2015; **12**(1): 19-23.
95. Botros RM, Sabry IM, Abdelbaky RS, et al. Vitamin D deficiency among healthy Egyptian females. *Endocrinol Nutr* 2015; **62**(7): 314-21.
96. El Maataoui A, El Maghraoui A, Biaz A, et al. Relationships between vertebral fractures, sex hormones and vitamin D in Moroccan postmenopausal women: a cross sectional study. *BMC Womens Health* 2015; **15**: 41.
97. Kazem YI, Moaty MIA, El-Shebini SM, et al. Low Vitamin D Serum Levels May Be a Modifiable Risk Factor for Obesity and Cognitive Impairment in Middle-Age Egyptian Women. *Open Access Macedonian Journal of Medical Sciences* 2014; **2**(2): 283-8.
98. Durazo-Arvizu RA, Camacho P, Bovet P, et al. 25-Hydroxyvitamin D in African-origin populations at varying latitudes challenges the construct of a physiologic norm. *Am J Clin Nutr* 2014; **100**(3): 908-14.
99. George JA, Micklesfield LK, Norris SA, et al. The association between body composition, 25(OH)D, and PTH and bone mineral density in black African and Asian Indian population groups. *J Clin Endocrinol Metab* 2014; **99**(6): 2146-54.
100. George JA, Norris SA, van Deventer HE, et al. Effect of adiposity, season, diet and calcium or vitamin D supplementation on the vitamin D status of healthy urban African and Asian-Indian adults. *Br J Nutr* 2014; **112**(4): 590-9.
101. Were T, Wesongah JO, Munde E, et al. Clinical chemistry profiles in injection heroin users from Coastal Region, Kenya. *BMC Clin Pathol* 2014; **14**(1): 32.
102. Aly WW, Hussein MA, Moahamed Ebeid S, et al. Prevalence of vitamin D insufficiency among community dwelling elderly in Dakahlia as a representative of rural areas in Egypt. *Aging Clin Exp Res* 2014; **26**(1): 47-51.
103. Durazo-Arvizu RA, Aloia JF, Dugas LR, et al. 25-hydroxyvitamin D levels in African American and Nigerian women. *Am J Hum Biol* 2013; **25**(4): 560-2.
104. Hamill MM, Ward KA, Pettifor JM, et al. Bone mass, body composition and vitamin D status of ARV-naive, urban, black South African women with HIV infection, stratified by CD(4) count. *Osteoporos Int* 2013; **24**(11): 2855-61.
105. George JA, Norris SA, van Deventer HE, et al. The association of 25 hydroxyvitamin D and parathyroid hormone with metabolic syndrome in two ethnic groups in South Africa. *PLoS One* 2013; **8**(4): e61282.
106. Gebreegziabher T, Stoecker BJ. Vitamin D insufficiency in a sunshine-sufficient area: southern Ethiopia. *Food Nutr Bull* 2013; **34**(4): 429-33.
107. Abu el Maaty MA, Hanafi RS, El Badawy S, et al. Association of suboptimal 25-hydroxyvitamin D levels with knee osteoarthritis incidence in post-menopausal Egyptian women. *Rheumatol Int* 2013; **33**(11): 2903-7.
108. Olama SM, Senna MK, Elarman MM, et al. Serum vitamin D level and bone mineral density in premenopausal Egyptian women with fibromyalgia. *Rheumatol Int* 2013; **33**(1): 185-92.
109. El-Shal AS, Shalaby SM, Aly NM, et al. Genetic variation in the vitamin D receptor gene and vitamin D serum levels in Egyptian women with polycystic ovary syndrome. *Mol Biol Rep* 2013; **40**(11): 6063-73.

110. Emerah AA, El-Shal AS. Role of vitamin D receptor gene polymorphisms and serum 25-hydroxyvitamin D level in Egyptian female patients with systemic lupus erythematosus. *Mol Biol Rep* 2013; **40**(11): 6151-62.
111. Friis H, Range N, Changalucha J, et al. Vitamin D status among pulmonary TB patients and non-TB controls: a cross-sectional study from Mwanza, Tanzania. *PLoS One* 2013; **8**(12): e81142.
112. Ibn Yacoub Y, Amine B, Laatiris A, et al. Bone density in Moroccan women with systemic scleroderma and its relationships with disease-related parameters and vitamin D status. *Rheumatol Int* 2012; **32**(10): 3143-8.
113. Schaalan MF, Mohamed WA, Amin HH. Vitamin D deficiency: correlation to interleukin-17, interleukin-23 and PIIINP in hepatitis C virus genotype 4. *World J Gastroenterol* 2012; **18**(28): 3738-44.
114. Luxwolda MF, Kuipers RS, Kema IP, et al. Traditionally living populations in East Africa have a mean serum 25-hydroxyvitamin D concentration of 115 nmol/l. *Br J Nutr* 2012; **108**(9): 1557-61.
115. Abd El Gawad SS, Abdul Samee ER, Metwali AA, et al. Vitamin D receptor gene polymorphism and its association with 1,25-dihydroxyvitamin D(3) in patients with Graves disease in an Egyptian population: a pilot study. *Endocr Pract* 2012; **18**(2): 132-9.
116. El Maghraoui A, Ouzzif Z, Mounach A, et al. Hypovitaminosis D and prevalent asymptomatic vertebral fractures in Moroccan postmenopausal women. *BMC Womens Health* 2012; **12**: 11.
117. Kruger MC, Kruger IM, Wentzel-Viljoen E, et al. Urbanization of black South African women may increase risk of low bone mass due to low vitamin D status, low calcium intake, and high bone turnover. *Nutr Res* 2011; **31**(10): 748-58.
118. Nansera D, Graziano FM, Friedman DJ, et al. Vitamin D and calcium levels in Ugandan adults with human immunodeficiency virus and tuberculosis. *Int J Tuberc Lung Dis* 2011; **15**(11): 1522-7, i.
119. Glew RH, Crossey MJ, Polanams J, et al. Vitamin D status of seminomadic Fulani men and women. *J Natl Med Assoc* 2010; **102**(6): 485-90.
120. Allali F, El Aichaoui S, Khazani H, et al. High prevalence of hypovitaminosis D in Morocco: relationship to lifestyle, physical performance, bone markers, and bone mineral density. *Semin Arthritis Rheum* 2009; **38**(6): 444-51.
121. Yan L, Schoenmakers I, Zhou B, et al. Ethnic differences in parathyroid hormone secretion and mineral metabolism in response to oral phosphate administration. *Bone* 2009; **45**(2): 238-45.
122. Wejse C, Olesen R, Rabna P, et al. Serum 25-hydroxyvitamin D in a West African population of tuberculosis patients and unmatched healthy controls. *Am J Clin Nutr* 2007; **86**(5): 1376-83.
123. Alsayed A, Gad A, Azab A. Serum 25-hydroxyvitamin D concentrations and metabolic syndrome in Egyptian men. *Journal of Medical Sciences* 2007; **7**(5): 850-4.
124. Meddeb N, Sahli H, Chahed M, et al. Vitamin D deficiency in Tunisia. *Osteoporos Int* 2005; **16**(2): 180-3.
125. Njemini R, Meyers I, Demanet C, et al. The prevalence of autoantibodies in an elderly sub-Saharan African population. *Clin Exp Immunol* 2002; **127**(1): 99-106.
126. Daniels ED, Pettifor JM, Moodley GP. Serum osteocalcin has limited usefulness as a diagnostic marker for rickets. *Eur J Pediatr* 2000; **159**(10): 730-3.
127. Daniels ED, Pettifor JM, Schnitzler CM, et al. Differences in mineral homeostasis, volumetric bone mass and femoral neck axis length in black and white South African women. *Osteoporos Int* 1997; **7**(2): 105-12.

128. Garabedian M, Ben-Mekhbi H. Is vitamin D-deficiency rickets a public health problem in France and Algeria? Nestle nutrition workshop series (USA); 1991; 1991.
129. Feleke Y, Abdulkadir J, Mshana R, et al. Low levels of serum calcidiol in an African population compared to a North European population. *Eur J Endocrinol* 1999; **141**(4): 358-60.
130. M'Buyamba-Kabangu JR, Fagard R, Lijnen P, et al. Calcium, vitamin D-endocrine system, and parathyroid hormone in black and white males. *Calcified tissue international* 1987; **41**(2): 70-4.
131. Pettifor JM, Ross FP, Moodley G, et al. Serum calcium, magnesium, phosphorus, alkaline phosphatase and 25-hydroxyvitamin D concentrations in children. *S Afr Med J* 1978; **53**(19): 751-4.
132. Viechtbauer W, Cheung MWL. Outlier and influence diagnostics for meta-analysis. *Research synthesis methods* 2010; **1**(2): 112-25.
133. Egger M, Davey Smith G, Schneider M, et al. Bias in meta-analysis detected by a simple, graphical test. *BMJ* 1997; **315**(7109): 629-34.
